# Transcriptome analysis and prediction of the metabolic state of stress-induced viable but non-culturable *Bacillus subtilis* cells

**DOI:** 10.1038/s41598-022-21102-w

**Published:** 2022-10-26

**Authors:** Luiza P. Morawska, Oscar P. Kuipers

**Affiliations:** Molecular Genetics Group, Groningen Biomolecular Sciences and Biotechnology Institute, University of Groningen, Nijenborgh 7, 9747 AG Groningen, The Netherlands

**Keywords:** Microbiology, Bacteria, Bacterial physiology, Antibacterial drug resistance

## Abstract

Many bacteria adapt their physiology and enter the viable but non-culturable state to survive prolonged exposure to adverse environmental conditions. The VBNC cells maintain active metabolism, membrane integrity and gene transcription. However, they lose the ability to form colonies on a conventional culture media. Thus, standard colony counting methods cannot detect these alive but dormant cells. The Gram-positive bacterium *Bacillus subtilis* was found to enter the VBNC state when pre-exposed to osmotic stress and treated with a lethal dose of kanamycin. These cells reduced their metabolic activity, ceased growth and division and became kanamycin-tolerant. Interestingly, despite active metabolism, the majority of the kanamycin tolerant cells could not be revived on LB agar. In this study, we use a robust RNA-Seq technique to elucidate the differences in transcriptional profiles of *B. subtilis* VBNC cells. A comparative analysis of differently expressed genes and operons performed in this study indicates high similarities in transcriptional responses of VBNC and kanamycin-sensitive cells to antibiotic treatment. Moreover, this work reveals that VBNC cells strongly upregulate genes involved in proline uptake and catabolism, suggesting a putative role of proline as nutrient in VBNC cells.

## Introduction

In their natural environment, microorganisms face many unfavorable conditions challenging their survival. To overcome potential threats, microorganisms can enter a new physiological dormant state known as the viable but non-culturable (VBNC) state. The VBNC state has been observed in many bacteria, including human pathogens, and proposed as one of survival mechanisms which allows cells to withstand long exposure to many adverse conditions^1,2^. Notably, VBNC cells share many characteristics with the persister cells^3^. Similarly to persisters, the VBNC cells are stochastically present in the population^4,5^, but can be also generated by environmental stresses^6–8^. Moreover, alike persisters, VBNC cells are metabolically active, however, they lose the ability to regrow on a medium that typically supports proliferation after the antibiotic pressure is removed^4,7,9–11^. While persisters can rapidly regrow on growth-promoting media, the VBNC cells require appropriate environmental stimuli or prolonged treatment to regain full culturability^11–16^. The similarities and co-occurrence of both phenotypes in the same culture suggested that these survival strategies are part of the dormancy continuum in which VBNC cells are in deeper dormancy state than persisters^4,17,18^.

One of the consequences of bacteria entering into the VBNC state is their tolerance to antibiotics increasing risk of reoccurring infections. Since antibiotics typically act on actively growing bacteria, non-dividing VBNC cells exhibiting low metabolic activity are less affected. Recent studies indicated the presence of antibiotic-resistant VBNC cells of several pathogens, including *Haemophilus influenzae*^19^, *Enterococcus faecalis*^20^, *Mycobacterium smegmatis*^21^, *Helicobacter pylori*^22^ and *Escherichia coli*^23^. Thus, identification of the mechanism of how bacteria enter the VBNC state is of high importance. In recent years, RNA sequencing (RNA-Seq) has been used to elucidate the mechanisms of the formation of various VBNC cells, including *E. coli*^24,25^, *Lactobacillus acetotolerans*^26^, *Rhodococcus biphenylivorans*^27^, and *Pseudomonas syringae*^28^. These studies demonstrated that changes in the gene expression of VBNC cells depend on the type of the inducer (stressor). The majority of studies, however, indicate common changes in transcriptome landscape of VBNC cells pointing to increased oxidative stress response, structural changes in the cell envelope, and reduced metabolic processes.

In our latest study, we showed that laboratory strain, *Bacillus subtilis* 168, enters the non-growing but metabolically active state when challenged with osmotic stress and becomes tolerant to lethal concentrations of kanamycin^29^. We demonstrated that pretreatment with hyper-osmotic stress, followed by transient hypo-osmotic stress and immediate kanamycin treatment locks down *B. subtilis* in a hyperpolarized state in which the cells still display metabolic activity and relatively high ATP levels. Importantly, “hibernated” cells could not proliferate when the antibiotic was removed from the environment, even after 20 h of incubation in a growth permissive medium. In this study, we attempt to characterize the transcriptional changes of *B. subtilis* in the VBNC state after 1 h of kanamycin treatment. With the use of RNA-Seq, we analyze differently expressed genes in VBNC *B. subtilis* cells induced by osmotic stress treatment to provide new insights into the VBNC state formation in sporulating bacteria.

## Results

### Global transcriptional profiles of *B. subtilis* under antibiotic stress

Our previous study showed that preadaptation of *B. subtilis* to transient osmotic upshift with 0.6 M NaCl protects cells when exposed to aminoglycosides^29^. We observed that the non-dividing preadapted cells enter the VBNC state and become insensitive to kanamycin when challenged with a lethal concentration of kanamycin. Here, we examined changes in the global gene expression profile of *B. subtilis* cells in the VBNC state with high-throughput RNA sequencing and compared the genome-wide transcriptional responses between studied conditions (Table 1).

**Table 1:** List of tested conditions.

Sample	Pretreatment condition	Condition after pretreatment
Control	90 min in SMM	1 h in SMM
Kanamycin	90 min in SMM	1 h in SMM + kanamycin 62.5 µg/ml
VBNC	90 min in 0.6 M NaCl SMM	1 h in SMM + kanamycin 62.5 µg/ml

The RNA of non-stressed culturable cells, kanamycin-sensitive and VBNC cells was subjected to RNA-seq, and the estimated RPKM (reads per kilobase per million reads mapped) values were evaluated using the T-REx pipeline^30^. The statistical analysis of gene expression data offers data normalization and determination of differently expressed genes (DEGs) between studied conditions—referred as contrasts in this publication.We noted that the library sizes and signal distributions were comparable for all samples (Fig. S1) and, therefore, sufficient to perform further differential gene expression (DGE) analysis.

**Figure 1:**
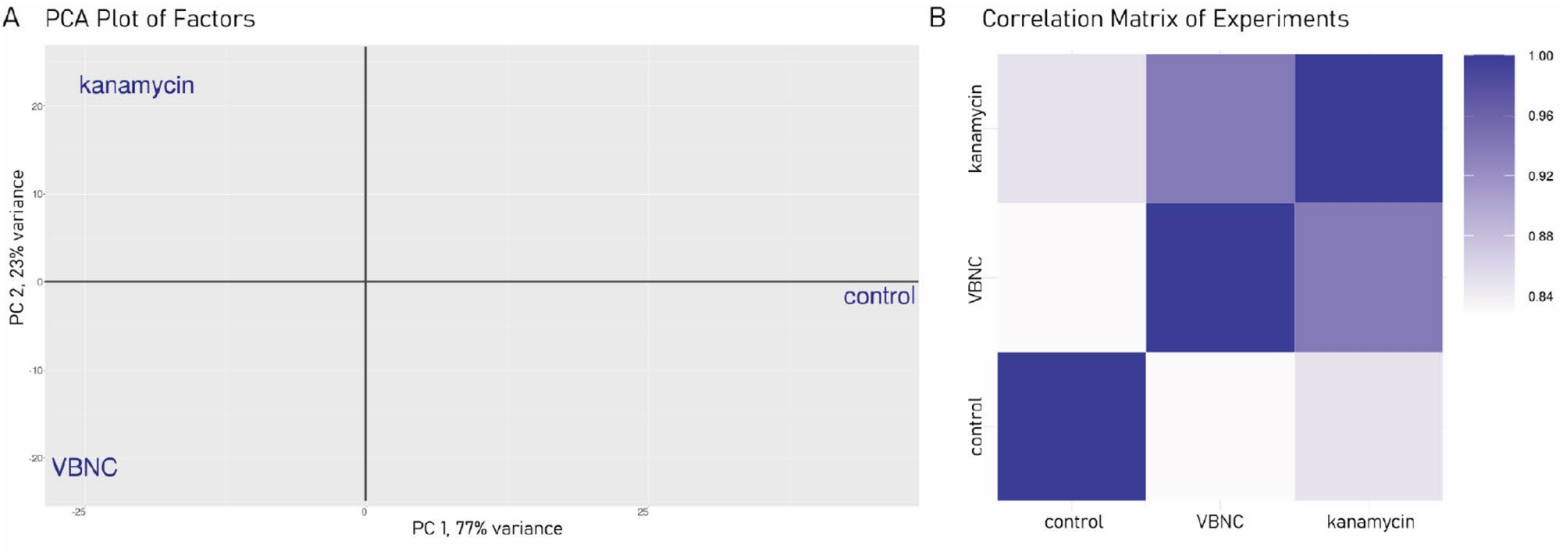
Similarities in the gene expression profiles across tested conditions. (**A**) PCA analysis. The gene expression for each condition represents an average of three biological samples. (**B**) Pearson’s correlation matrix of the same conditions as in (A). The maximum correlation value is 1 (including self-correlation). The correlation values for the three conditions ranged from 0.83 to 1.

To describe general differences in the global gene expression between experiments, we performed principal component analysis (PCA). PCA revealed that all three experiments were statistically well-distributed, indicating significantly different transcription profiles (Fig. 1A). Notably, VBNC and kanamycin samples displayed similar variance in the principal component 1 (PC1), indicating expression similarities for the genes within 77% variance; this may be due to the robust general and antibiotic stress response activation in antibiotic-stressed cells. The similarities between kanamycin-sensitive and VBNC cells transcriptomes were also observed with the correlation matrix of experiments (Fig. 1B).

In line with PCA and Pearson’s correlation matrix data (Fig. 1), the analysis of DEGs showed that the most substantial alteration of the gene expression was caused by kanamycin in the media. For the kanamycin-sensitive cells, which were not pretreated with high salt, a total of 388 and 394 genes were respectively significantly up- or downregulated when compared to the control conditions without kanamycin (kanamycin vs. control). Similarly, for the VBNC cells (VBNC vs. control), we noted 334 upregulated and 514 downregulated genes when compared to the untreated cells. Interestingly, the differences in gene expression between VBNC and kanamycin-sensitive cells (VBNC vs. kanamycin)were rather moderate, and revealed 73 upregulated and 185 downregulated genes in VBNC cells compared to kanamycin-sensitive ones. (Table 2).

**Table 2:** Differently expressed genes in tested conditions.

Contrasts	HighFold (log_2_FC ≥ 5, *p* ≤ 0.01)		TopHits (log_2_FC ≥ 2, *p* ≤ 0.05)		Total affected genes	
	Upregulated	Downregulated	Upregulated	Downregulated	Upregulated	Downregulated
Kanamycin versus control	13	9	375	385	388	394
VBNC versus control	10	22	330	492	334	514
VBNC versus kanamycin	9	2	64	182	73	185

### Functional analysis of DEGs of *B. subtilis* cells in the VBNC state

**Figure 2:**
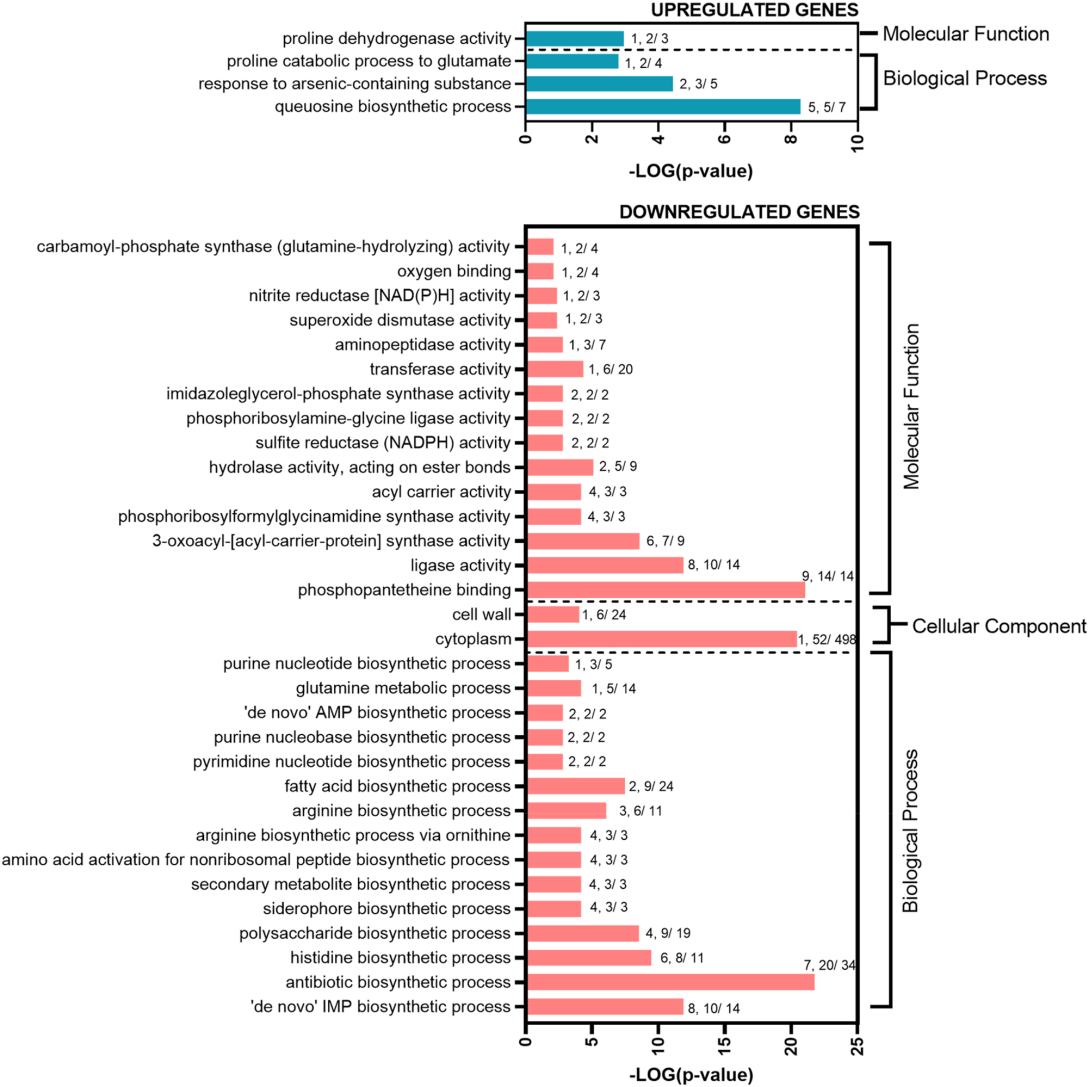
GO terms significantly affected in *B. subtilis* VBNC cells. A graphical representation of DEGs (*p* value ≤ 0.05 and a log_2_FC ≥ 3) annotated by GO analysis in GSEA-Pro3. The values given to each bar correspond to Score (0–9, 0 being not significant, ranked based on Benjamini–Hochberg Algorithm) and the hits/class size.

For functional interpretation of DGEs of VBNC cells, we performed Gene Set Enrichment Analysis (GSEA). Statistically significant differences in gene expression (*p* value ≤ 0.05 and a log_2_FC ≥ 3) were categorized into functional classes and annotated by Gene Ontology (GO) analysis using FUNAGE-Pro v1 software^31^. To determine the differences between VBNC and culturable state (VBNC vs. control), a total of 114 upregulated and 213 downregulated genes (Table S1) were additionally mapped to operons, and the most relevant functions are discussed below. The overrepresented GO terms are depicted in Fig. 2.

#### Significantly upregulated genes in *B. subtilis* VBNC cells

**Figure 3:**
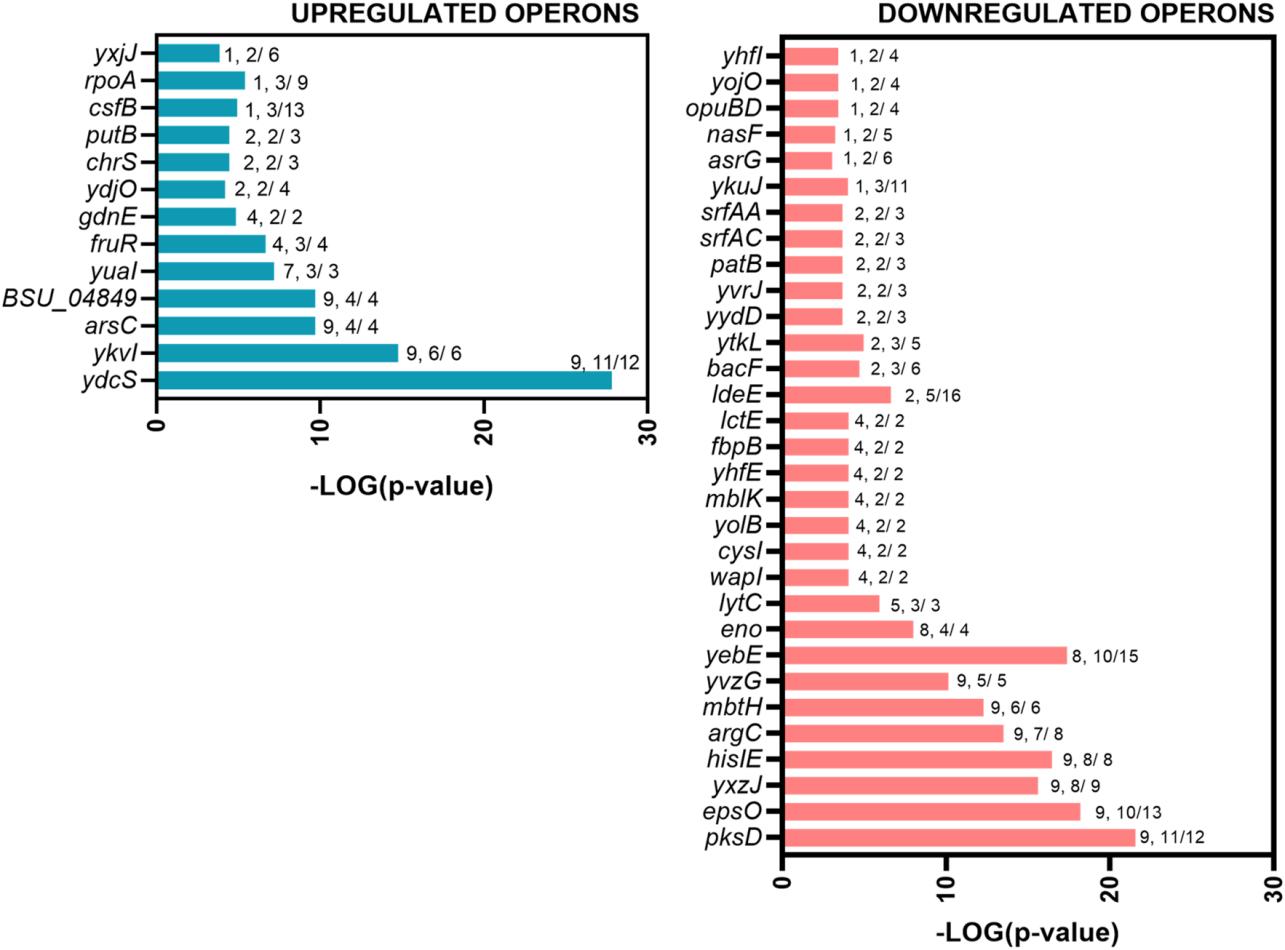
Significantly affected gene expression organized in operons for *B. subtilis* VBNC cells. A graphical representation of DEGs (*p* value ≤ 0.05 and a log_2_FC ≥ 3) clustered in operons. The values given to each bar correspond to Score (0–9, 0 being not significant, ranked based on Benjamini–Hochberg Algorithm) and the hits/class size.

The analysis of DEGs revealed the presence of four highly enriched GO terms in VBNC cells (Fig. 2). More detailed GSEA demonstrated that VBNC cells highly upregulate 14 different operons compared to the non-stressed culturable cells (VBNC vs. control) (Fig. 3). Interestingly, the most prominent changes were observed for ICE*Bs1* conjugative element genes of *ydcS* and *BSU_04849* operons (*yddJ, yddI, cwlT, conG, yddF, conE, conD, conC, conB, yddA, ydcT, ydcS, nicK, conQ, helP, xis*). The induction of ICE*Bs1* is controlled by quorum sensing mechanism (induced at high population densities)^32^ or stressed-induced DNA damage and SOS response^33^. Because VBNC cells cease their growth and thus do not reach high densities, we suggest that the activation of ICE*Bs1* excision likely resulted in response to antibiotic-induced oxidative stress and DNA damage^34,35^.

**Figure 4:**
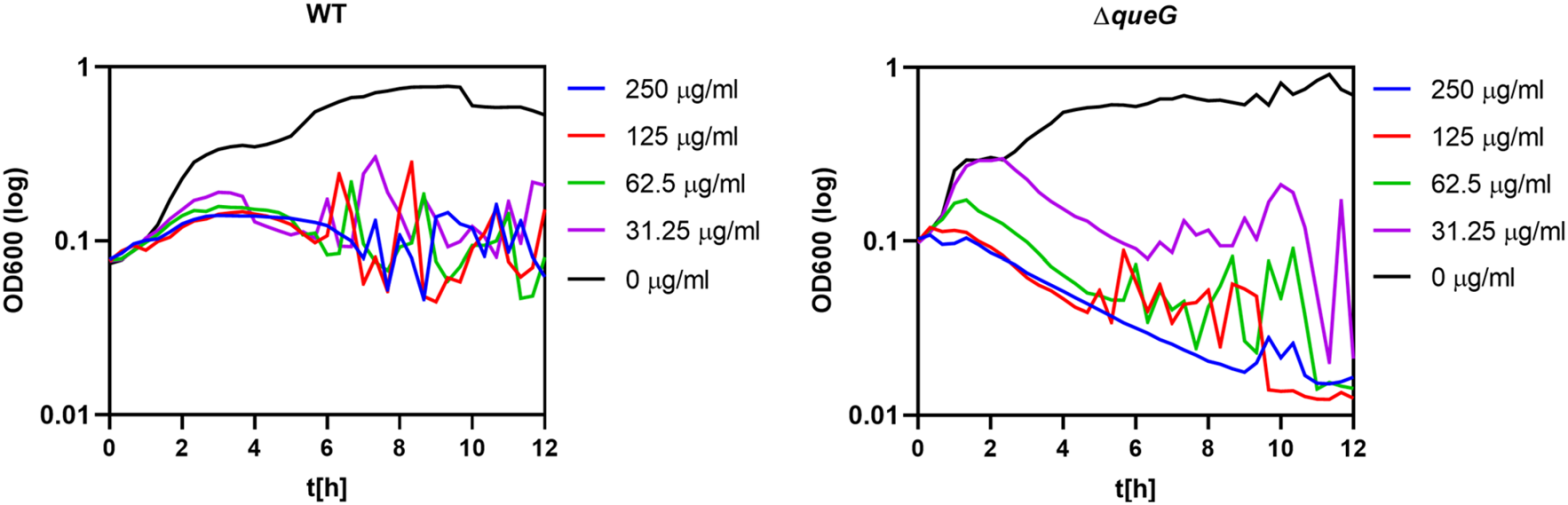
Susceptibility of *B. subtilis* 168 ∆*queG* mutant to lethal concentrations of kanamycin. Growth curves for WT *B. subtilis* 168 and ∆*queG* in the presence of increasing concentrations of kanamycin. The black curve indicates the outgrowth of cells without antibiotics.

The second most significantly upregulated genes in VBNC cells belong to the queosine biosynthesis pathway, particularly the *queC-queD-queE-queF* operon (*yvkI*), regulated by the preQ-riboswitch^36,37^ (Fig. 3). Queuosine (Q) is a modified nucleotide synthesized de novo from GTP and is typically found in the wobble position of anticodons of four tRNA species in both eukaryotes and prokaryotes (His, Asp, Asn and Tyr)^38^. Interestingly, the latest studies have shown that eukaryotic Q-modified tRNAs are involved in the nutritional control of protein synthesis by controlling the translational speed of Q-decoded codons^39^. In the absence of queuine, cells displayed increased protein unfolding and activated protein-folding stress responses. Since kanamycin binds to the 30S ribosomal subunit and causes protein misfolding, we believe that VBNC cells increase queuosine pools and Q-modified tRNAs levels to minimize the translation error and thereby confer the kanamycin-tolerant phenotype. Prompted by this observation, we tested whether the mutation of the *queG* gene encoding for the last enzymatic step of the conversion of epoxyqueuosine to queuosine^40^ would potentiate the kanamycin-induced cell death in *Bacillus subtilis* 168. The exponentially growing cells of *B. subtilis* 168 ∆*queG* were transferred to media with increasing concentrations of kanamycin and we followed their outgrowth for 12 h (Fig. 4). Initially, up to 2 h of the antibiotic treatment, the *queG* mutant showed increased growth rates when compared to the wild type strain under all tested concentrations. This increased growth rate was likely caused by the decreased intracellular levels of queuosine in the mutant strain. After 2 h, however, we observed a substantial decrease in the optical density of the ∆*queG* strain and more prominent antibiotic efficacy compared to the wild type strain.

The adverse effects of antibiotic-induced oxidative stress were evident with the increased expression of operons related to detoxification of inorganic ions, including *arsR-yqcK-arsB-arsC* operon and members of *chrS-ywrB-ywrA* operon. The bactericidal killing mechanism has been correlated with the induced formation of reactive oxygen species (ROS)^41–43^, which oxidize cellular structures, including sulfur-containing amino acids and metal-containing cofactor sites in proteins^44^. This leads to protein unfolding and accumulation of free metal ions in the cytoplasm and metal stress^45^. Interestingly, metal efflux genes were also highly upregulated in *E.coli* VBNC cells induced in low-level chlorination^25^ and low-temperature conditions^46^.

Furthermore, we observed that VBNC cells activate the expression of a SigW-controlled operon *yuaF-floT-yuaI*, suggesting changes in membrane composition and fluidity of cells exposed to kanamycin^47,48^. The overexpression of *yuaI operon* suggests that membrane of antibiotic-treated cells is more rigid due to enrichment in FloT and YuaF proteins^48^, and thereby, less permeable for membrane-interacting cationic antibiotics like kanamycin^49,50^.

**Figure 5:**
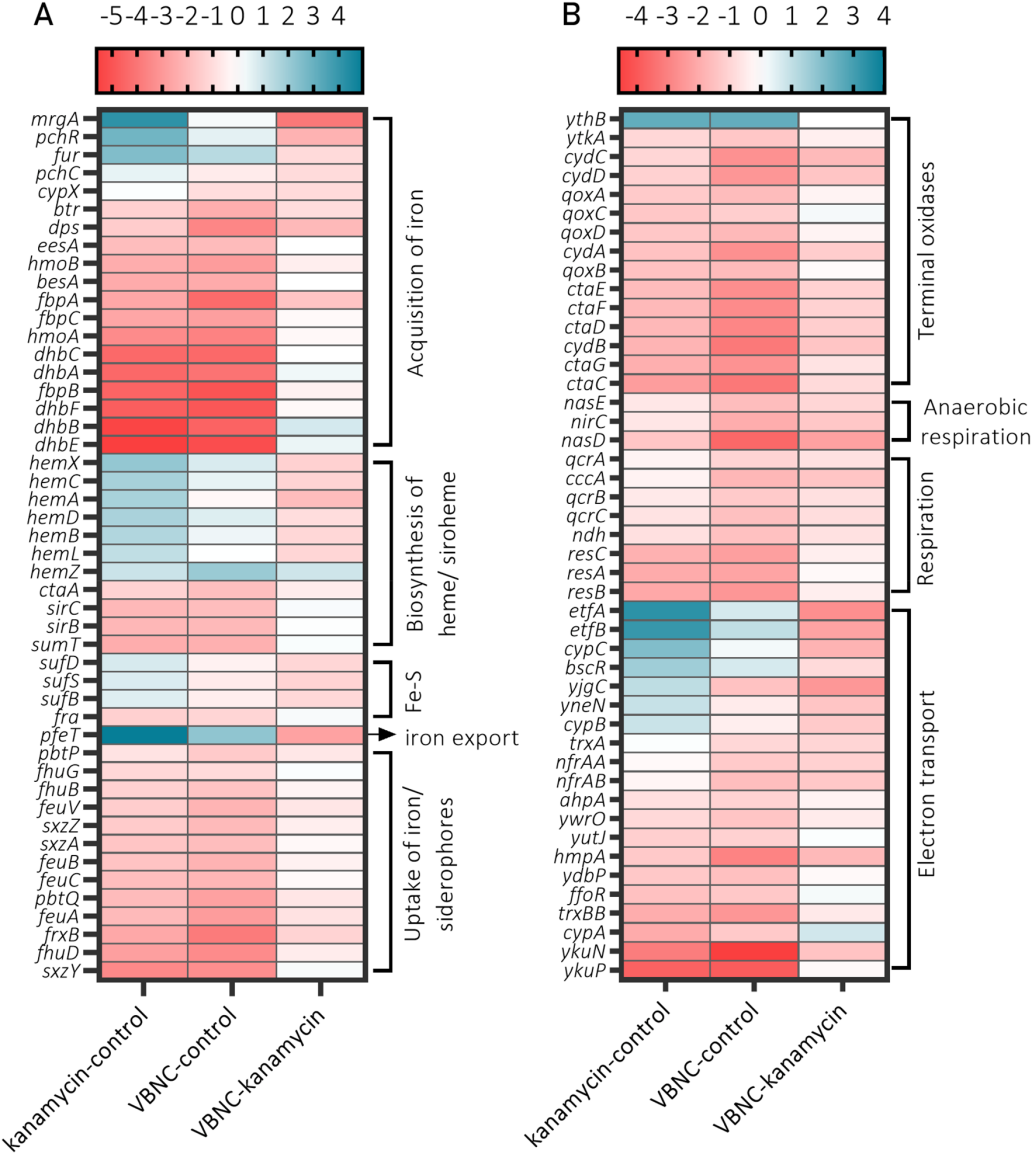
TopHit DEGs involved in iron homeostasis and respiration in *B. subtilis* VBNC and kanamycin-sensitive cells. Heatmaps of DGEs ratio values (*p* value ≤ 0.05 and a log_2_FC ≥ 2) involved in (**A**) iron homeostasis and (**B**) respiration in *B. subtilis* 168. The analysis shows significantly different expressed genes in at least one of the experimental contrasts. The fold differences are represented with the color bar above each heatmap. Heatmaps were generated in GraphPad Prism 8 Software (GraphPad Software, San Diego, California USA, www.graphpad.com).

In this study, the VBNC cells emerged in SMM media supplemented with glucose as a primary carbon source. Interestingly, transcriptomics data indicated that *B. subtilis* in the VBNC state activates the transcription of *fruR-fruK-fruA* operon (Fig. 5, *fruR*) and switches to fructose utilization. Moreover, the overrepresentation of proline utilization genes in *putB* operon (*putB* and *putC*) indicates a secondary use of proline as a carbon and nitrogen source. Since the VBNC state in *B. subtilis* was induced with a transient osmotic upshift, the utilization of accumulated and released to the media proline is expected^51^. Moreover, previous studies have shown that *B. subtilis* also upregulates *putB* and *putC* in response to lack of glucose in the media^52^. Thus, it appears that *B. subtilis* VBNC cells avoid metabolizing highly-energetic glucose and switch to alternative carbon sources to maintain basal metabolic activity.

#### Significantly downregulated genes in *B. subtilis* VBNC cells

For the highly downregulated genes, the GO analysis revealed that cells in the VBNC state arrest many anabolic processes and cell division, presumably to reduce energy consumption dedicated to the growth and to enter dormancy (Fig. 2). The enrichment analysis showed 31 operons significantly affected in VBNC cells when compared to culturable cells (VBNC vs. control) (Fig. 3).

Many downregulated operons were classified to the AbrB regulon, controlled with AbrB transcription factor^53–55^. The most significant changes were found for operons associated with biosynthesis of secondary metabolites, including nonribosomal peptides like bacillibactin, plipastatin, bacilysin and surfactin (*mbtH*, *ldeE*, *bacF*, *sfrAC, sfrAA*), and polyketides (*pksD*), as well as operons involved in biofilm formation (*epsO*) and motility and swarming (*yvzG*). Some other AbrB-controlled operons, involved in competition with other bacteria in biofilms (*arsG*)^56,57^, expression of extracellular signal peptides (*yolB*)^58^, cell separation (part of *yojO* operon, *cwlS* gene)^59^ were also strongly downregulated. The downregulation of AbrB-controlled genes suggest that VBNC cells are likely locked in transition phase between active growth and commitment to sporulation^60^.

VBNC cells are in a non-growing transition state, therefore changes in the expression of genes involved in central metabolism and cell growth are expected. In this regard, our data revealed that VBNC cells displayed significantly lower transcription levels of several glycolytic genes, including *eno*, *gpmI*, *tpiA* and *pgk* (*eno* operon)*,* as well as genes involved in histidine and arginine biosynthesis (*hisIE*, *argC*, *ytkL*). Moreover, VBNC cells significantly downregulated genes involved in purine biosynthesis (*yebE* operon), corresponding with low metabolic activity^61^ and reduced histidine biosynthesis^62^. Additionally, the analysis showed downregulation of N-acetylmuramoyl-L-alanine amidases genes *lytA-lytC* and dl-endopeptidases genes *lytD, lytF* and *cwlS*, responsible for cell wall turnover and cell separation^63^, and downregulation of the members of *mblK* operon associated with the control of cell shape and division (*yisK*)^64^, strongly point to reduced synthesis and disassembly of peptidoglycan and inhibited cell proliferation.

Finally, the analysis of transcription profiles of VBNC and culturable cells showed changes for genes involved in response to iron limitation. The downregulation of several members of Fur-regulated operons *fbpB dhbB*, *ykuJ* and *nasF* indicates strong inhibition of iron-scavenging components and reduced nitrate assimilation, most likely due to the increased pools of free iron and oxidative stress in VBNC cells^65–67^. The analysis of TopHits data (*p* value ≤ 0.05 and a log_2_FC ≥ 2) additionally confirmed that VBNC cells activate the iron export (*pfeT*) and downregulate the genes involved in iron acquisition (Fig. 5A). Changes in the expression of genes involved in the respiration show that VBNC cells shut down most of the respiration systems with an exception of *ythB* an alternative terminal quinol oxidase^68,69^ and *etfAB* encoding electron transfer flavoprotein involved in fatty acid β-oxidation^70^ (Fig. 5B).

### Differences in transcription profile of VBNC and kanamycin-sensitive cells

Our previous study showed that *B. subtilis* VBNC cells, induced by transient treatment with a non-lethal osmotic upshift, are resistant to kanamycin^29^. Hence, to gain more insights into the possible resistance mechanism, we analyzed the differences in transcriptomes of VBNC cells and kanamycin-sensitive cells after 1 h of the antibiotic treatment (VBNC vs. kanamycin). As previously observed, after 1 h treatment with kanamycin the kanamycin-sensitive population was still alive, therefore the isolation of total RNA and RNA-Seq were performed on antibiotic-affected but still viable cells^29^. Accordingly, we performed the GSEA for DEGs and ascribed the most overrepresented genes into GO terms and operons. Since the global analysis of DEGs for both conditions showed moderate changes in gene expression when compared (Fig. 1), we adjusted the cutoff value to a *p* value ≤ 0.05 and a log_2_FC ≥ 2 (TopHits). Thus, a total of 246 DEGs were identified, including 64 upregulated and 182 downregulated genes (Table S2).

**Figure 6:**
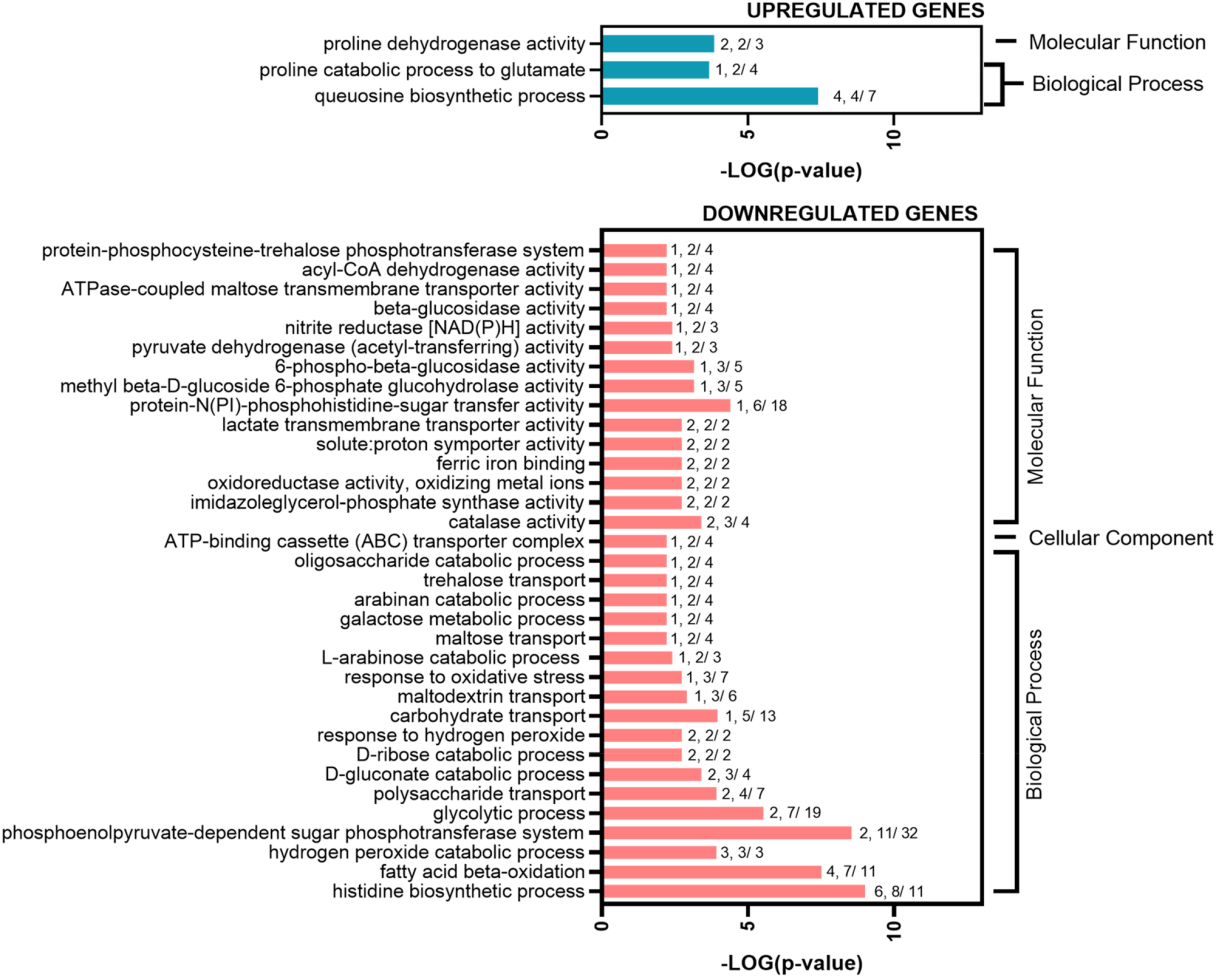
GO terms significantly affected in *B. subtilis* VBNC cells versus kanamycin-sensitive cells. A graphical representation of DEGs (*p* value ≤ 0.05 and a log_2_FC ≥ 2) annotated by GO analysis. The values given to each bar correspond to Score (0–9, 0 being not significant, ranked based on Benjamini–Hochberg Algorithm) and the hits/class size.

The GO analysis of significantly affected DEGs revealed that VBNC cells downregulate several biological processes compared with kanamycin-sensitive *B. subtilis* (Fig. 6). Most GO terms identified for VBNC cells point to repressed transport and catabolism of alternative carbon sources, downregulation of processes involved in glycolysis and oxidative stress response, and increased queuosine biosynthesis and proline catabolism.

**Figure 7:**
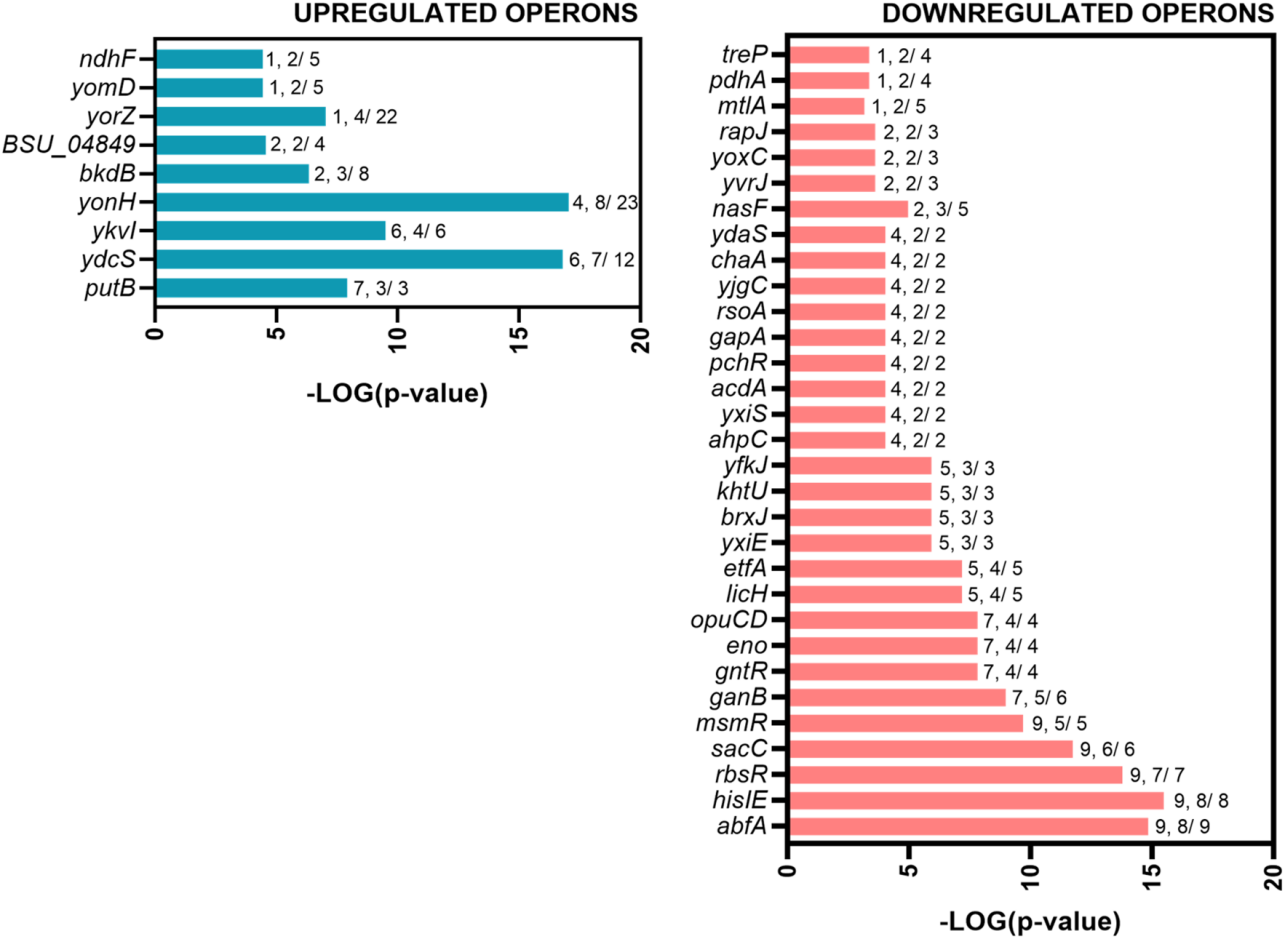
Significantly affected operons in *B. subtilis* VBNC cells compared to kanamycin-sensitive cells. A graphical representation of DEGs (*p* value ≤ 0.05 and log_2_FC ≥ 2) clustered in operons. The values given to each bar correspond to Score (0–9, 0 being not significant, ranked based on Benjamini–Hochberg Algorithm) and the hits/class size.

The GSEA demonstrated 9 upregulated and 31 downregulated operons in VBNC cells compared to the kanamycin-sensitive cells (VBNC vs. kanamycin) (Fig. 7). Members of 9 upregulated operons are primarily related to proline utilization (*putB*), conjugation of ICE*Bs1* element (*ydcS*, *BSU_04849*), synthesis of the modified ribonucleotide queuosine (*ykvI*) and lysogeny of SP-beta prophage (*yonH*, *yorZ*, *yomD*). Interestingly, we identified that genes *buk*, *bcd* and *ptb* (*bkdB* operon), involved in the catabolism of branched-chain amino acids (BCAAs), i.e. leucine, valine and isoleucine are highly upregulated in the VBNC cells^71^. Importantly, the deamination and oxidative decarboxylation of BCAAs generate precursors for biosynthesis of fatty acid species and determine the composition of the cytoplasmatic membrane^72–74^. We did not observe significant differences in the expression of genes in *bkdB* operon in VBNC cells when compared with the control. Thus, we propose that kanamycin-sensitive cells downregulate genes involved in BCAAs degradation and likely utilize fatty acids in response to aminoglycoside stress (increased level of *fadE*, *fadA*, *fadN* transcripts in the kanamycin sensitive cells Table S2).

Furthermore, the upregulation of members of *ndhF* operon (i.e. *ndhF* and *ybcI*) suggests a potential mechanism maintaining the membrane potential and regulating the intracellular pH in VBNC cells via translocation of cations such as H^+^ and Na^+^^75^.

For the highly downregulated genes in VBNC cells, the most significant changes were identified for operons involved in sugar transport and utilization, including arabinose (*abfA*), ribose (*rbsR*), levan (*sacC*), melobiose (*msmR*), galactan (*ganB*), gluconate (*gntR*), lichenan (*licH*), mannitol (*mtlA*) and trehalose (*treP*), indicating strong activation of secondary sugar metabolism in kanamycin-sensitive cells in contrast to VNBC cells (Fig. 7). Furthermore, downregulation of genes in *opuCD* and *khtU* operons pointed to limited uptake of compatible solutes like glycine betaine, and efflux of potassium ions in VBNC cells compared to kanamycin-sensitive cells, showing that antibiotic-stressed cells likely suffer turgor loss. This is with accordance to recent work of Wong et al. 2021 proposing that bactericidal antibiotics induce cell death and lysis by inducing cytoplasmic condensation through membrane damage^76^. Since cytoplasmic condensation and lysis are associated with loss of cellular turgor^77^, the initial increase of potassium pools and import of compatible solutes in kanamycin-sensitive cells could possibly offset the high ionic strength of the cytoplasm^78–80^.

**Figure 8:**
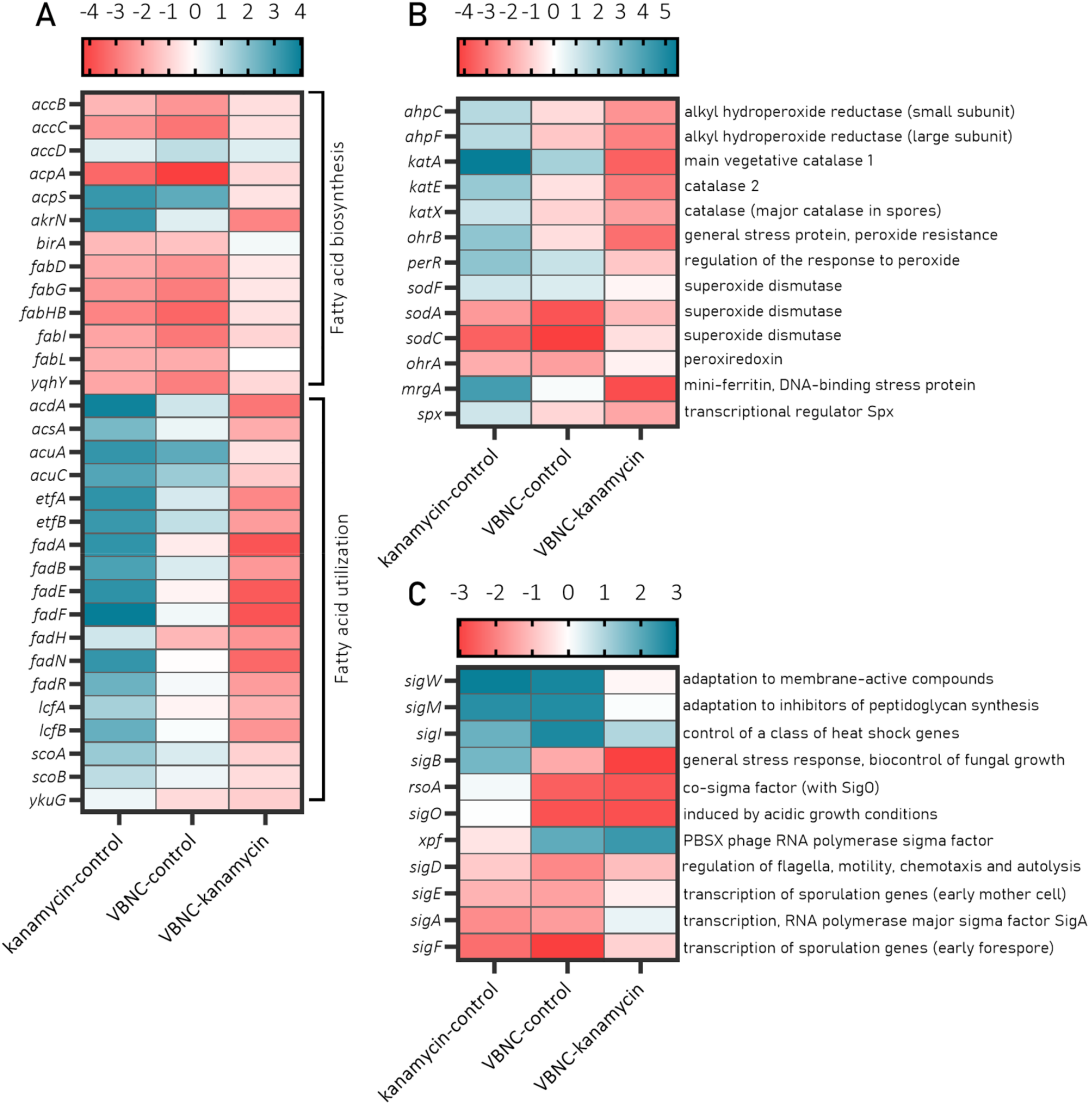
TopHit DEGs for genes involved in fatty acid biosynthesis, oxidative stress and transcription factors in *B. subtilis* VBNC and kanamycin-sensitive cells. Heatmaps of DGEs ratio values (*p* value ≤ 0.05 and a log_2_FC ≥ 2) for (**A**) fatty acid biosynthesis genes, (**B**) genes encoding for oxidative agents detoxification and (**C**) expression levels of transcription factors. The analysis shows significant changes in the gene expression in at least one of the experimental contrasts. The fold change indication is presented above the heatmaps. Heatmaps were generated in GraphPad Prism 8 Software (GraphPad Software, San Diego, California USA, www.graphpad.com).

**Figure 9:**
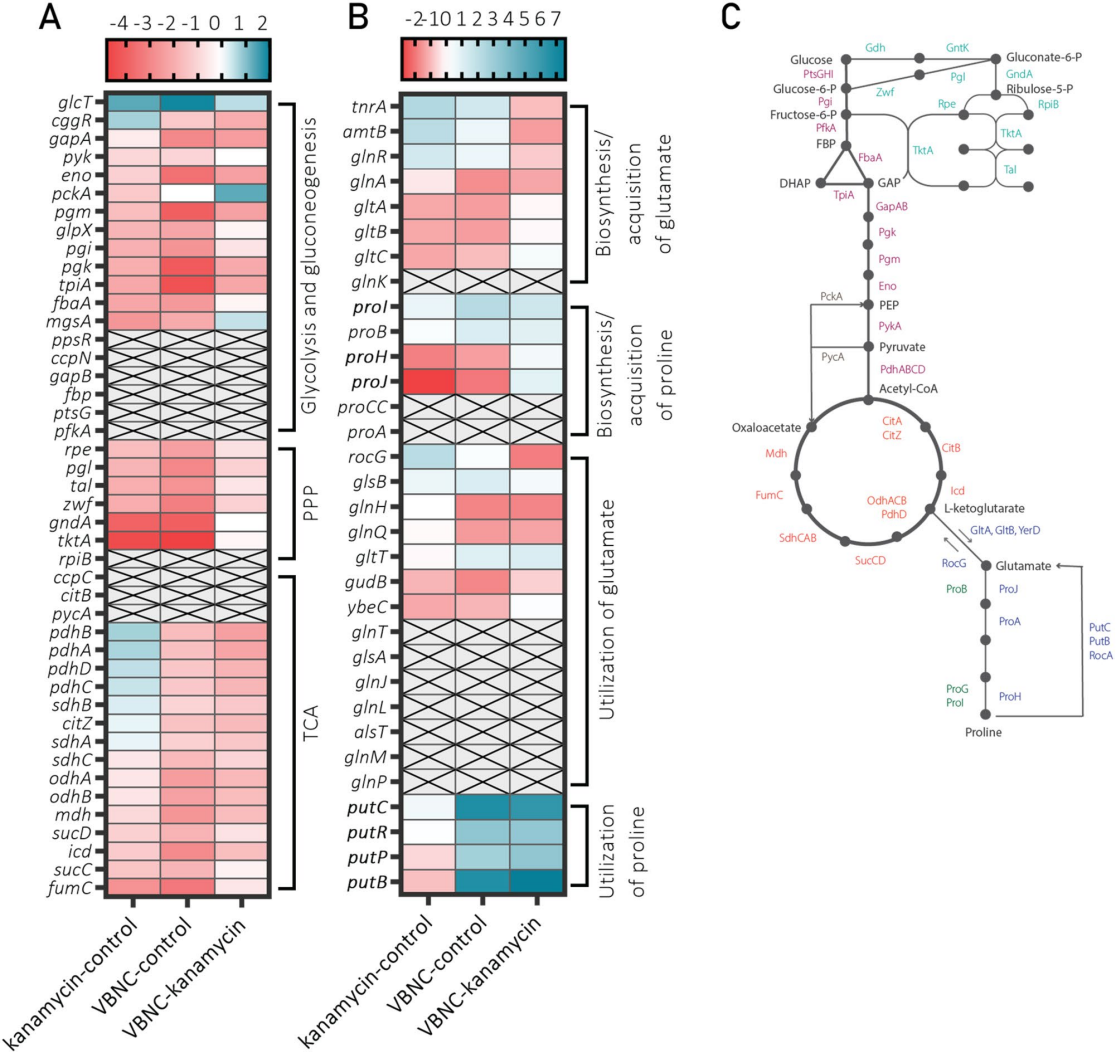
Changes in central metabolism in VBNC cells versus kanamycin-sensitive cells. Heatmaps of TopHits of DGEs ratio values (*p* value ≤ 0.05 and a log_2_FC ≥ 2) for (**A**) central metabolism genes and (**B**) proline and glutamate metabolism. (**C**) depicts the TCA cycle, glycolysis, PPP and proline metabolism in *B. subtilis* 168. Heatmaps were generated in GraphPad Prism 8 Software (GraphPad Software, San Diego, California USA, www.graphpad.com).

The analysis of downregulated operons confirmed that cells respond to aminoglycoside treatment by upregulating genes for fatty acid utilization (*eftA* and *acdA* operon) in the first hour of antibiotic exposure. However, the response of VBNC cells seems to be less severe compared to the kanamycin-sensitive cells (VBNC vs. kanamycin) (Fig. 8A). Furthermore, differences in central metabolism between VBNC and kanamycin-sensitive cells showed strong downregulation of glycolytic operons *eno*, *gapA* and *pdhA* in VBNC cells (Fig. 7). The analysis of individual genes revealed that in VBNC cells most of the genes related to central metabolism are significantly downregulated compared to kanamycin-sensitive cells with an exception for the ones encoding for proline catabolism enzymes (Fig. 9). Taken all together, it could be hypothesized that VBNC cells enter into the glucose starvation-like mode and utilize not only alternative carbohydrates but also reach for fatty acids and proline as an energy source.

Remarkably, genes involved in general and oxidative stress response were significantly downregulated in VBNC cells (*brxJ*, *yfkJ*, *ahpC*, *yxiS*, *pchR*, *rsoA*, *yjgC*, *ydaS*, *yvrJ*, *yoxC* and *rapJ* operons) (Fig. 7). Detailed analysis of DEGs showed that genes encoding several catalases, including main catalase KatA, superoxide dismutases, SodA and SodC, and alkyl hydroperoxide reductases, AhpC and AhpF were significantly downregulated in VBNC cells when compared to kanamycin-sensitive and/or culturable cells (Fig. 8B). Decreased induction of H_2_O_2_ detoxifying enzymes in VBNC cells shows that despite kanamycin being present inside the cell, the antibiotic-induced oxidative damage has been reduced. This is in accordance to our last work on ROS formation in VBNC and kanamycin-sensitive cells showing that VBNC cells display decreased intracellular ROS pools likely due to limited respiration. In *B. subtilis*, the expression of oxidative stress-related genes is regulated by three transcription factors PerR, OhrR and SigB^81^. Our data shows that in VBNC cells, genes encoding for SigB and PerR are downregulated, whereas the changes in the *ohrR* transcription levels were not significant between studied conditions (Fig. 8B,C). Collectively, the transcriptomic profile of VBNC cells revealed that VBNC cells are less affected by kanamycin stress compared to the kanamycin-sensitive cells.

## Discussion

In this study, we attempted to elucidate the underlying mechanism of *B. subtilis* cells entering the VBNC state using robust RNA-Seq method, focusing on global changes in the transcriptome of VBNC cells compared to culturable cells with and without 1 h of kanamycin stress (VBNC vs. control). The comparative analysis of DEGs revealed significant changes in several processes in *B. subtilis* VBNC cells compared to the culturable cells. The strong overrepresentation of conjugative element ICE*Bs*1 and SPβ prophage transcripts, increased expression of inorganic ions exporters, and downregulation of iron scavenging components indicate the presence of severe oxidative stress and activation of the SOS-response in VBNC cells. The induction of oxidative stress might occur initially due to the pretreatment with osmotic stress and be further potentiated upon the antibiotic treatment. Since aminoglycoside-mediated cell death has been correlated with increased ROS accumulation in aerobic conditions^41,82^, increased pools of inorganic ions, including toxic ferric ions, could arise from ROS-induced damage of iron-sulfur clusters. Another indicator of ROS-mediated cell damage is increased expression of main catalase KatA, superoxide dismutase SodF, and transcriptional regulator of genes involved in peroxide stress PerR in VBNC cells. Notably, when compared with kanamycin-sensitive cells (VBNC vs. control), the expression of oxidative stress-induced genes in VBNC cells was significantly lower. Although VBNC cells accumulate kanamycin similarly to kanamycin-sensitive cells, their biochemical response to the antibiotic seems to be less severe, supporting our last observations on ROS levels in VBNC and kanamycin-sensitive cells^29^.

Our recent work demonstrated that *B. subtilis* VBNC cells induced due to the changes in the environment’s osmolarity are metabolically active, however, their metabolic activity was substantially reduced compared to the culturable cells^29^. The RNA-Seq analysis supported our latest study and revealed significant downregulation of genes involved in glycolysis, TCA cycle and PPP, suggesting that VBNC cells suppress most of the genes of central metabolism. Notably, VBNC cells, as well as kanamycin-sensitive cells (VBNC vs. kanamycin), displayed increased expression of genes involved in utilization of secondary carbohydrates. This indicates that kanamycin-treated cells avoid consumption of highly energetic carbohydrates when challenged with kanamycin, and relieve carbon catabolite repression, entering a glucose starvation-like mode. Interestingly, only VBNC cells displayed a high upregulation of genes involved in proline uptake and utilization. Since VBNC cells were induced with the osmotic stress, the cells synthesized adequate amount of proline to maintain cells’ turgor^51^. We propose, that accumulated, secreted and recaptured from the media proline is utilized by VBNC cells to support the basal metabolism.

Another indication of the reduced metabolic activity and decelerated translation rates was the downregulation of the synthesis of purine and pyrimidine nucleotides and amino acids, including arginine and histidine. The limited pools of nucleotides and amino acids may halt cell growth and division and result in the loss of culturability in the VBNC cells. Similar observations regarding nucleotide metabolism and amino acid synthesis were made for *E.coli* VBNC cells induced by high-pressure CO_2_^24^.

Moreover, our study indicated for the first time that antibiotic-treated cells likely modify the translation rates by increasing the Q-modified tRNAs and limiting mistranslation, similarly to the mechanisms proposed in eukaryotic cells, where Q-modified tRNAs were shown to be involved in the nutritional control of protein synthesis by controlling the translational speed of Q-decoded codons^39^. The DEGs analysis revealed increased levels of queuosine biosynthesis genes transcripts in both VBNC and kanamycin-sensitive cells, suggesting that *B. subtilis* exposed to kanamycin increase the Q-tRNAs pools to slow down the translation rates. Interestingly, the mutation in the *queG* gene encoding for the last enzymatic step for conversion of epoxyqueuosine to queuosine^40^ indeed potentiated kanamycin-induced cell death.

VBNC cells are locked in a non-dividing state, hence, changes in the expression of genes involved in cell envelope processes are to be expected. Notably, *sigM* and *sigW* encoding transcriptional factors for genes involved in adaptation to membrane-active compounds and inhibitors of peptidoglycan synthesis were highly upregulated when cells were challenged with kanamycin (Fig. 8C). Moreover, the downregulation of N-acetylmuramoyl-L-alanine amidases and dl-endopeptidases suggests that kanamycin-treated cells cease synthesis and disassembly of the peptidoglycan and halt the cell wall elongation and division^63^.

Concluding, we defined the transcriptional profiles of *B. subtilis*’s VBNC and kanamycin-sensitive cells which revealed high similarities in regard to induction of stress response related genes, downregulation of genes involved in metabolism and translation, and induction of genes related to cell envelope turnover. We were able to identify specific traits displayed by VBNC cells, including activation of proline catabolism and strong upregulation of queuosine biosynthesis genes. Future work will focus on exploring the role of proline and tRNA modifications in kanamycin tolerant VBNC cells.

## Experimental procedures

### Bacterial strains, culture conditions and media

*B. subtilis* 168 laboratory strain was grown in Spizizen’s Minimal Medium^83^ (SMM) supplemented with 0.5% (w/v) glucose, 1% (v/v) trace elements. To maintain controlled growth conditions, 3-day cultivation was performed as follows: *B. subtilis* was streaked on selective LB agar plates and incubated overnight (O/N) at 37 °C. The next morning, 3 ml liquid LB was inoculated with a single colony and incubated for 8 h at 37 °C and 220 rpm. After the incubation period, the culture was 1000-fold diluted in SMM medium and grown O/N at 37 °C and 220 rpm. The overnight culture was diluted to OD600 of 0.08 in fresh SMM medium and grown until the OD600 reached a value of 0.3. Exponentially growing cultures of *B. subtilis* 168 in SMM were equally distributed to 3 flasks and subjected to different preconditions.

### VBNC state induction in *B. subtilis* 168

To induce the VBNC state in *B. subtilis* 168, the exponentially growing culture was stressed with 0.6 M NaCl for 90 min. After 90 min, the preadapted with salt cells were centrifuged at 4000 × g for 2 min at room temperature (RT). The cell pellets were immediately resuspended in NaCl-free SMM containing 62,5 µg/ml of kanamycin and grown for 1 h at 37 °C and 220 rpm. For control samples, the cells were incubated for 90 min in SMM without 0.6 M NaCl. Subsequently, cells were collected, and pellets were resuspended in fresh SMM with or without 62,5 µg/ml kanamycin. Control samples were incubated for 1 h at 37 °C and 220 rpm, similarly to induced VBNC cultures. After 1 h, cells were harvested by centrifugation at 10.000 × g for 1 min, snap-frozen in liquid nitrogen and stored at − 80 °C prior to RNA isolation.

### RNA isolation

Collected pellets were resuspended in 200 µl of TE buffer, and 50 ul of 10% SDS and 0.25 g of glass beads were added. The cells were disrupted by two 1 min cycles in a Biospec Mini-BeadBeater (Biospec Products, US) with 1 min incubation on ice in between cycles. To fully lyse the cells, 0.5 ml of TRIzol (TRIzol™ Reagent, Invitrogen) was added, and samples were vortexed and incubated at 70 °C for 20 min. Afterwards, 100 µl of chloroform was added, and samples were vortexed for 15 s and incubated for 2 min at RT. Followed by a centrifugation step (12.500 × g, 15 min, 4 °C), the aqueous phase (~ 300 µl) was transferred to a clean Eppendorf tube with 2 volumes of 100% ethanol. RNA precipitation was performed with 1:1 (v/v) isopropanol extraction and 20 mg/ml of glycogen. Samples were vortexed for 10 s and incubated at RT for 10 min. After centrifugation (12.000 × g, 5 min, 4 °C), the supernatant was removed, and the RNA pellet was washed with 100 µl of 70% ice-cold ethanol. Next, samples were centrifuged at 12.000 × g for 5 min at 4 °C and ethanol was removed, and RNA pellets were air-dried for 5 min. RNA was eluted with 30 µl of nuclease-free milli-Q water, and the quality was assessed by gel electrophoresis. RNA samples were stored at − 80 °C prior to sequencing.

### RNA sequencing and data analysis

Before sequencing, rRNA depletion and cDNA preparation was performed using Zymo-Seq RiboFree Total RNA-Seq Library Kit (Zymo Research, cat. 3003). The cDNA libraries were sequenced on Illumina NextSeq550. FastQ files were mapped to the *B. subtilis* 168 reference genome (GCA_000009045.1) using Bowtie2 and the RPKM (Reads Per Kilobase per Million reads) values were used for T-REx analysis pipeline. The statistical analysis was performed with T-REx software and GSEA-Prov3 for the gene enrichment analysis.

## Supplementary Information

Supplementary Information.

## Data Availability

The RNA-Seq data has been deposited in the NCBI GEO database. GEO accession number: GSE207180 (https://www.ncbi.nlm.nih.gov/geo/query/acc.cgi?acc=GSE207180).
