# Supplementary material for: Transcriptome analysis and prediction of the metabolic state of stress-induced viable but non-culturable Bacillus subtilis cells
Source: Sci Rep. 2022 Oct 26;12:18015. doi: 10.1038/s41598-022-21102-w (PMC9605947; doi:10.1038/s41598-022-21102-w)
Supplement: Supplementary file 1 — Supplementary Information. [file 41598_2022_21102_MOESM1_ESM.pdf]

Transcriptome analysis and prediction of the metabolic state of stress-induced viable but non-culturable *Bacillus subtilis* cells

Luiza P. Morawska[1], Oscar P. Kuipers[1]*

[1]Molecular Genetics Group, Groningen Biomolecular Sciences and Biotechnology Institute, University of Groningen, Nijenborgh 7, 9747 AG Groningen, Netherlands

*corresponding author

o.p.kuipers@rug.nl

SUPPLEMENTARY MATERIALS

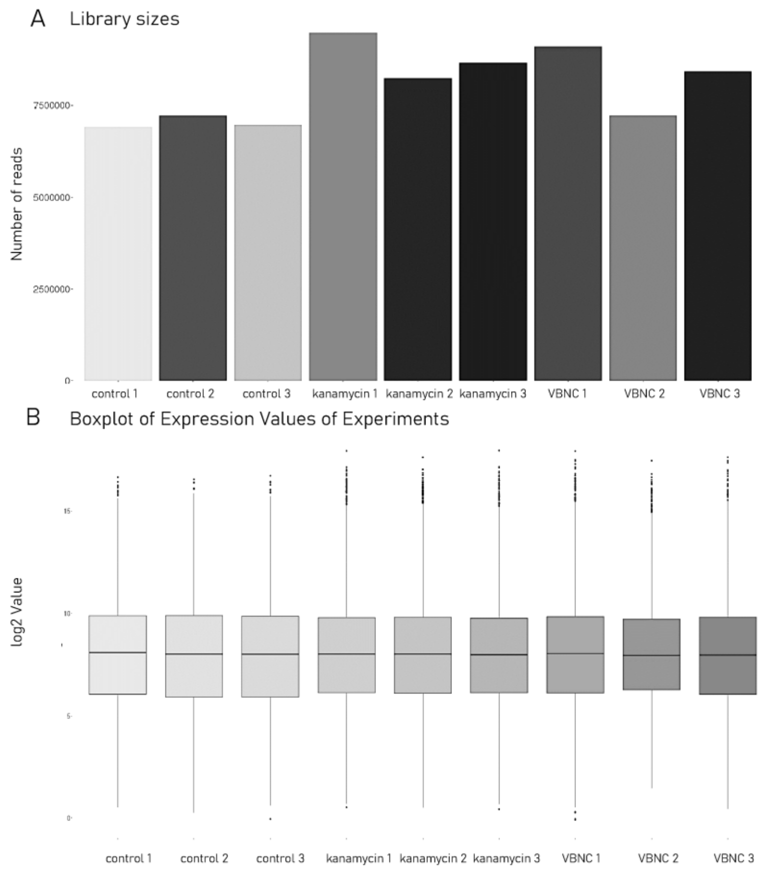

Figure S1. Distribution of reads across tested conditions.
(**A**) total number of RNA reads, (**B**) box-plots of normalized signals per condition (Multi-Dimensional Scaling plot).

Table S1. Significantly upregulated and downregulated genes in the VBNC cells compared to the culturable cells (p-value ≤ 0.05 and a log2FC ≥ 3)

Locus tag	Gene	Fold change	Function
BSU_13700	*clpE*	8.7	ATP-dependent Clp protease (class III stress gene)
BSU_03210	*putC*	6.6	1-pyrroline-5-carboxylate dehydrogenase
BSU_03200	*putB*	6.6	proline oxidase

BSU_09040	*yhcD*	5.9	hypothetical protein
BSU_27190	*yrzI*	5.8	conserved protein of unknown function
BSU_04900	*yddA*	5.3	*ICEBs1* mobile element: conserved protein of unknown function
BSU_13520	*ykrP*	5.3	putative integral membrane protein putative acyltransferase
BSU_13720	*queC*	5.2	pre-queuosine 0 synthase
BSU_26619	*yrzO*	5.1	conserved protein of unknown function
BSU_27185	*BSU_27185*	5.1	hypothetical protein
BSU_13740	*queE*	5.0	7-carboxy-7-deazaguanine synthase
BSU_13730	*queD*	4.8	6-carboxy-5-tetrahydropterin synthase queuosine biosynthesis
BSU_misc_RNA_22	*qswA*	4.7	
BSU_12519	*yjzJ*	4.7	phage PBSX conserved hypothetical protein
BSU_19290	*yozO*	4.6	conserved hypothetical phage protein
BSU_26610	*yrkA*	4.5	putative membrane associated protein
BSU_04920	*conC*	4.5	*ICEBs1* mobile element: subunit of the conjugation machinery
BSU_27935	*BSU_27935*	4.5	hypothetical protein
BSU_25780	*arsC*	4.5	thioredoxin-coupled arsenate reductase skin element
BSU_04890	*ydcT*	4.5	*ICEBs1* mobile element: conserved protein of unknown function
BSU_misc_RNA_80	*sdaM*	4.4	
BSU_05610	*vmlR*	4.4	ATP-binding cassette efflux transporter
BSU_12069	*yjzH*	4.4	conserved hypothetical protein
BSU_14390	*fruK*	4.4	fructose-1-phosphate kinase
BSU_14400	*fruA*	4.3	phosphotransferase system (PTS) fructose-specific enzyme
BSU_04970	*cwlT*	4.3	two-domain autolysin with N-acetylmuramidase and DL-endopeptidase activity
BSU_05470	*mneP*	4.2	primary Mn(II) efflux pump
BSU_13750	*queF*	4.2	NADPH-dependent 7-cyano-7-deazaguanine reductase
BSU_18580	*yoaF*	4.2	conserved protein of unknown function
BSU_04980	*yddI*	4.2	*ICEBs1* mobile element: conserved protein of unknown function
BSU_04880	*ydcS*	4.2	*ICEBs1* mobile element: conserved protein of unknown function
BSU_19260	*yocM*	4.2	putative chaperone
BSU_20570	*yoqN*	4.1	consserved hypothetical protein%3B phage SPβ
BSU_ncRNA_1	*aimX*	4.1	small RNA controlling lysogeny of phage SPβ
BSU_26310	*sknH*	4.1	skin element%3B factor binding to DnaA
BSU_31830	*yueE*	4.0	putative metal-dependent phosphohydrolase
BSU_35510	*yvyE*	4.0	putative translation regulator
BSU_misc_RNA_8	*aswA*	4.0	
BSU_12229	*yjzI*	4.0	putative phage protein
BSU_14380	*fruR*	4.0	transcriptional regulator (DeoR family)
BSU_06290	*yeaA*	3.9	conserved hypothetical protein
BSU_04910	*conB*	3.9	*ICEBs1* mobile element: subunit of the conjugation machinery
BSU_35130	*yvlA*	3.9	conserved protein of unknown function
BSU_36090	*ywrE*	3.9	conserved protein of unknown function
BSU_24200	*yqiH*	3.9	putative lipoprotein
BSU_04870	*nicK*	3.9	*ICEBs1* mobile element: DNA relaxase
BSU_13330	*ykoL*	3.9	conserved protein of unknown function
BSU_04960	*conG*	3.8	*ICEBs1* mobile element: VirB6 subunit of the conjugation machinery
BSU_00250	*xpaC*	3.8	putative phosphatase
BSU_06280	*ydjP*	3.8	putative aminoacrylate hydrolase
BSU_24190	*yqiI*	3.8	N-acetylmuramoyl-L-alanine amidase
BSU_06320	*mneS*	3.8	secondary Mn(II) exporter
BSU_04860	*conQ*	3.8	*ICEBs1* mobile element: coupling conjugation protein VirD4
BSU_25810	*arsR*	3.7	transcriptional regulator (ArsR-arsenate skin element
BSU_18980	*yobJ*	3.7	conserved protein of unknown function, putative defective prophage 6
BSU_17370	*nrdI*	3.7	co-factor of ribonucleotide diphosphate reductase
BSU_31020	*nfeDB*	3.7	putative membrane integrity integral membrane protein
BSU_36350	*ywpD*	3.7	putative two-component sensor histidine kinase
BSU_14620	*slp*	3.7	small peptidoglycan-associated lipoprotein
BSU_04470	*dctP*	3.6	C4-dicarboxylate transport protein
BSU_25490	*hrcA*	3.6	transcriptional regulator of heat-shock genes
BSU_01880	*ybcI*	3.6	conserved hypothetical protein, prophage 1 region
BSU_25790	*arsB*	3.6	arsenite efflux transporter, skin element
BSU_29040	*ytbD*	3.6	putative transporter
BSU_17840	*fosB*	3.5	magnesium-dependent bacillithiol-transferase
BSU_17920	*spo0D*	3.4	Spo0A-P phosphatase
BSU_21420	*bhlA*	3.4	holin-like protein, bacteriophage SPβ
BSU_33460	*yvgT*	3.4	putative integral membrane protein

BSU_misc_RNA_60	*gdwB*	3.4	
BSU_30010	*ythP*	3.4	putative ABC transporter (ATP-binding protein)
BSU_25800	*yqcK*	3.4	putative thiol lyase
BSU_38940	*yxjI*	3.4	conserved protein of unknown function
BSU_00240	*csfB*	3.4	forespore-specific anti-sigma factor
BSU_39750	*iolB*	3.4	5-deoxy-D-glucuronic acid isomerase
BSU_03230	*putR*	3.4	transcriptional activator of proline degradation operon
BSU_38018	*ywzG*	3.3	putative transcriptional regulator, PadR family
BSU_23190	*dacB*	3.3	D-alanyl-D-alanine carboxypeptidase
BSU_08540	*yfhI*	3.3	putative efflux transporter
BSU_misc_RNA_61	*mswI*	3.3	
BSU_38840	*gdnE*	3.3	guanidinium exporter
BSU_04930	*conD*	3.3	*ICEBs1* mobile element: subunit of the conjugation machinery
BSU_31010	*floT*	3.3	flotillin lipid rafts scaffold protein
BSU_26550	*yrkD*	3.3	putative metal-sensitive transcriptional regulator involved in sulfur metabolism
BSU_26449	*BSU_26449*	3.3	hypothetical protein
BSU_18400	*yoeD*	3.3	putative excisionase
BSU_12420	*yjoB*	3.2	informational ATPase possibly involved in protein degradation
BSU_26935	*BSU_26935*	3.2	hypothetical protein
BSU_31820	*yuzF*	3.2	conserved protein of unknown function
BSU_13710	*ykvI*	3.2	putative transporter
BSU_04850	*helP*	3.2	*ICEBs1* mobile element: helicase processivity factor
BSU_07620	*yflN*	3.2	putative metal-dependent hydrolase
BSU_31000	*yuaI*	3.2	putative N-acetyltransferase
BSU_01450	*ecfA*	3.2	energizing coupling factor of ABC influx transporter (ATP-binding protein)
BSU_25440	*rsmE*	3.1	methylase of U1498 in 16S rRNA
BSU_17630	*yncC*	3.1	putative sugar transporter
BSU_36120	*chrB*	3.1	chromate transporter subunit N
BSU_36110	*chrS*	3.1	transcriptional negative regulator of chromate transport (Lrp/AsnC family)
BSU_01460	*ecfAB*	3.1	energizing coupling factor of ABC influx transporter (ATP-binding protein)
BSU_39010	*rlmA*	3.1	23S rRNA m(1)G748 methyltransferase (class II)
BSU_28830	*ysdB*	3.1	conserved protein of unknown function
BSU_00260	*yaaN*	3.1	putative toxic compound resistance protein
BSU_18596	*BSU_18596*	3.1	conserved hypothetical protein
BSU_04940	*conE*	3.1	*ICEBs1* mobile element: VirB4-like ATPase
BSU_04950	*yddF*	3.1	*ICEBs1* mobile element: conserved protein of unknown function
BSU_13670	*mhqR*	3.0	transcriptional regulator (MarR family)
BSU_14009	*ykzT*	3.0	hypothetical protein
BSU_misc_RNA_4	*ldlM*	3.0	
BSU_misc_RNA_83	*tboY*	3.0	
BSU_04590	*ydbS*	3.0	resistance to heterologous antibiotics
BSU_08910	*queG*	3.0	epoxyqueuosine reductase
BSU_04849	*BSU_04849*	3.0	*ICEBs1* mobile element: hypothetical protein
BSU_17300	*ebrA*	3.0	small toxic metabolite efflux transporter subunit
BSU_19250	*yocL*	3.0	conserved protein of unknown function
BSU_39700	*iolG*	3.0	myo-inositol 2-dehydrogenase/D-chiro-inositol 3-dehydrogenase
BSU_37360	*sboX*	-7.2	putative bacteriocin-like product
BSU_11799	*yjzK*	-6.9	conserved hypothetical protein
BSU_misc_RNA_75	*bsrF*	-5.7	
BSU_07480	*yfmG*	-5.7	putative enzyme
BSU_03780	*phrC*	-5.7	secreted regulator of the activity of phosphatase RapC and competence and sporulation stimulating factor (CSF)
BSU_11240	*carB*	-5.6	arginine-specific carbamoyl-phosphate synthetase (large subunit)
BSU_03520	*srfAD*	-5.5	surfactin synthetase
BSU_11250	*argF*	-5.4	ornithine carbamoyltransferase
BSU_34920	*hisG*	-5.4	ATP phosphoribosyltransferase
BSU_19410	*cwlS*	-5.4	peptidoglycan hydrolase (cell wall-binding d,l-endopeptidase)
BSU_13180	*metE*	-5.3	cobalamin-independent methionine synthase
BSU_34910	*hisD*	-5.3	histidinol dehydrogenase
BSU_39220	*wapI*	-5.3	antitoxin of WapA tRNase
BSU_03510	*srfAC*	-5.2	surfactin synthetase
BSU_11230	*carA*	-5.2	arginine-specific carbamoyl-phosphate synthetase (small subunit)
BSU_31980	*dhbE*	-5.2	2,3-dihydroxybenzoate-AMP ligase
BSU_03490	*srfAB*	-5.2	surfactin synthetase

BSU_29450	*argG*	-5.1	argininosuccinate synthase
BSU_29440	*argH*	-5.1	argininosuccinate lyase
BSU_03480	*srfAA*	-5.0	surfactin synthetase
BSU_04530	*fbpB*	-5.0	regulator of iron homeostasis
BSU_34890	*hisH*	-5.0	imidazole glycerol phosphate synthase, glutamine amidotransferase subunit
BSU_34900	*hisB*	-5.0	imidazoleglycerol-phosphate dehydratase [Mn(II)-dependent]
BSU_34930	*hisZ*	-5.0	histidyl-tRNA synthetase-like subunit of ATP phophoribosyltransferase
BSU_34870	*hisF*	-4.9	imidazole glycerol phosphate synthase subunit
BSU_31960	*dhbF*	-4.9	siderophore, bacillibactin synthetase
BSU_34860	*hisIE*	-4.9	bifunctional phosphoribosyl-AMP cyclohydrolase
BSU_11220	*argD*	-4.9	N-acetylornithine aminotransferase
BSU_33240	*oxdC*	-4.9	oxalate decarboxylase
BSU_07560	*pelA*	-4.9	pectate lyase
BSU_39230	*wapA*	-4.8	cell wall-associated tRNA nuclease precursor, intercellular growth inhibitor
BSU_13950	*mcpC*	-4.8	methyl-accepting chemotaxis protein
BSU_17890	*tktA*	-4.8	transketolase
BSU_19745	*BSU_19745*	-4.8	conserved hypothetical protein
BSU_06490	*purF*	-4.7	glutamine phosphoribosylpyrophosphate amidotransferase
BSU_37460	*rapF*	-4.7	response regulator aspartate phosphatase anti-activator of ComA
BSU_35350	*yvyC*	-4.7	putative flagellar protein of unknown function
BSU_35630	*lytB*	-4.7	modifier of major autolysin amidase LytC
BSU_11210	*argB*	-4.6	N-acetylglutamate 5-phosphotransferase (acetylglutamate kinase)
BSU_17160	*pksH*	-4.6	enoyl-CoA hydratase for bacillaene biosynthesis
BSU_17200	*pksM*	-4.6	trans AT polyketide synthase of type I involved in bacillaene synthesis
BSU_35620	*lytC*	-4.6	N-acetylmuramoyl-L-alanine amidase (major autolysin)
BSU_31970	*dhbB*	-4.6	isochorismatase (siderophore specific)
BSU_14150	*fldN*	-4.6	short-chain flavodoxin (acts in lipid desaturation)
BSU_11200	*argJ*	-4.6	ornithine acetyltransferase amino-acid acetyltransferase
BSU_23980	*artP*	-4.5	high affinity arginine ABC transporter binding lipoprotein
BSU_34880	*hisA*	-4.5	phosphoribosylformimino-5-aminoimidazole carboxamide ribotide isomerase
BSU_17190	*pksL*	-4.5	polyketide synthase of type I (bacillaene synthesis)
BSU_14160	*ykuO*	-4.5	conserved hypothetical protein
BSU_06500	*purM*	-4.5	phosphoribosylaminoimidazole synthetase
BSU_33239	*yvrJ*	-4.5	factor involved in oxalate decarboxylase expression
BSU_40660	*yybF*	-4.4	putative permease
BSU_31990	*dhbC*	-4.4	isochorismate synthase (siderophore-specific)
BSU_06520	*purH*	-4.4	inosine-monophosphate cyclohydrolase
BSU_33920	*tpiA*	-4.4	triose phosphate isomerase
BSU_31080	*bslA*	-4.4	biofilm hydrophobic layer component
BSU_04536	*fbpA*	-4.4	regulator of iron homeostasis
BSU_12410	*yjoA*	-4.3	putative DNA-binding protein
BSU_06510	*purN*	-4.3	phosphoribosylglycinamide formyltransferase
BSU_19400	*sodC*	-4.3	superoxide dismutase (exported lipoprotein)
BSU_11040	*yitM*	-4.3	conserved hypothetical protein
BSU_17170	*pksI*	-4.3	decarboxylase involved in bacillaene synthesis
BSU_15920	*acpA*	-4.3	acyl carrier protein
BSU_21540	*yolA*	-4.3	conserved exported protein of unknown function, SPβ phage
BSU_11190	*argC*	-4.3	N-acetylglutamate gamma-semialdehyde dehydrogenase
BSU_35640	*lytA*	-4.3	membrane bound lipoprotein
BSU_21530	*yolB*	-4.2	conserved protein of unknown function, phage SPβ
BSU_18320	*ppsC*	-4.2	non-ribosomal plipastatin synthetase C involved in synthesis of plipastatin
BSU_06530	*purD*	-4.2	phosphoribosylglycinamide synthetase
BSU_33400	*pgoN*	-4.2	promiscuous glyoxal/methylglyoxal reductase
BSU_37710	*bacD*	-4.2	alanine-anticapsin ligase
BSU_18978	*bsrE*	-4.2	type I toxin (BsrE/AsrE)
BSU_17180	*pksJ*	-4.2	polyketide synthase of type I involved in nonribosomal synthesis of bacillaene
BSU_33930	*pgk*	-4.2	phosphoglycerate kinase
BSU_29430	*ytzD*	-4.1	conserved protein of unknown function
BSU_32000	*dhbA*	-4.1	2,3-dihydro-2,3-dihydroxybenzoate dehydrogenase
BSU_37700	*bacE*	-4.1	efflux protein for bacilysin excretion, self-protection against bacilysin
BSU_24790	*yqgX*	-4.1	putative metal-binding hydrolase
BSU_40420	*purA*	-4.1	adenylosuccinate synthetase
BSU_06480	*purL*	-4.1	phosphoribosylformylglycinamidine synthetase subunit II
BSU_17210	*pksN*	-4.0	trans AT polyketide synthase of type I involved in bacillaene synthesis
BSU_28820	*frvX*	-4.0	putative fructose-lysine aminopeptidase

BSU_18540	*yoaB*	-4.0	negatively charged metabolite transporter
BSU_32040	*hisP*	-4.0	histidine / basic amino acid transporter
BSU_33910	*pgm*	-4.0	phosphoglycerate mutase
BSU_23860	*gndA*	-4.0	NADP+-dependent 6-P-gluconate dehydrogenase
BSU_26210	*yqaR*	-4.0	hypothetical protein, skin element
BSU_06460	*purS*	-4.0	factor required for phosphoribosylformylglycinamidine synthetase activity
BSU_17150	*pksG*	-4.0	acetyl-S-AcpK β-ketothioester bacillaene intermediate transferase
BSU_22600	*aroA*	-4.0	3-phosphoshikimate 1-carboxyvinyltransferase
BSU_40200	*yydD*	-4.0	putative DNA wielding protein
BSU_14170	*fldP*	-3.9	short-chain flavodoxin
BSU_misc_RNA_82	*surF*	-3.9	
BSU_39610	*frxB*	-3.9	desferrioxamine-and ferrichrome-binding transporter lipoprotein
BSU_37690	*bacF*	-3.9	phenylalanine aminotransferase forming tetrahydrotyrosine in bacilysin synthesis
BSU_39210	*yxiF*	-3.9	conserved protein of unknown function
BSU_37660	*pta*	-3.9	phosphotransacetylase
BSU_18310	*ppsD*	-3.9	nonribosomal plipastatin synthetase D
BSU_10180	*yhfC*	-3.9	putative integral membrane protein
BSU_35319	*yvzG*	-3.9	conserved protein of unknown function
BSU_31959	*mbtH*	-3.8	stimulator of DhbF tyrosine adenylation activity
BSU_25020	*sodA*	-3.8	superoxide dismutase (Mn[2+]-dependent)
BSU_02700	*estA*	-3.8	secreted alkaliphilic lipase
BSU_33710	*opuBC*	-3.8	choline ABC transporter (choline-binding lipoprotein)
BSU_00490	*spoVG*	-3.8	regulator required for spore cortex synthesis (stage V sporulation)
BSU_34230	*epsN*	-3.8	putative aminotransferase involved in biofilm matrix formation
BSU_18330	*ppsB*	-3.8	nonribosomal plipastatin synthetase B involved in synthesis of plipastatin
BSU_10380	*hemAT*	-3.8	heme-based dioxygen sensor
BSU_21940	*degR*	-3.8	activator of degradative enzymes (aprE, nprE, sacB) production or activity
BSU_18300	*ppsE*	-3.8	nonribosomal plipastatin synthetase E
BSU_40210	*yydC*	-3.8	conserved hypothetical protein
BSU_20850	*aimP*	-3.8	arbitrium lysis /lysogeny regulatory peptide, phage SPβ
BSU_35340	*fliD*	-3.8	flagellar hook-associated capping protein 2
BSU_00730	*cysK*	-3.7	cysteine synthase
BSU_17130	*acpK*	-3.7	acyl-carrier protein involved in bacillaene synthesis
BSU_07150	*hmoA*	-3.7	heme-degrading monooxygenase
BSU_06470	*purQ*	-3.7	phosphoribosylformylglycinamidine synthetase subunit I
BSU_34340	*epsD*	-3.7	putative extracellular matrix glycosyltransferase
BSU_03300	*nasD*	-3.7	assimilatory nitrite reductase subunit
BSU_34290	*epsI*	-3.7	putative polysaccharide pyruvyl transferase involved in biofilm matrix formation
BSU_35310	*hpf*	-3.6	ribosome dimerisation factor
BSU_35330	*fliS*	-3.6	flagellar assembly protein FliS
BSU_21546	*bsrG*	-3.6	phage toxin, type I toxin-antitoxin system
BSU_11010	*samT*	-3.6	bifunctional homocysteine S-methyltransferase
BSU_38920	*pepT*	-3.6	peptidase T (tripeptidase)
BSU_05329	*ydzO*	-3.6	hypothetical protein
BSU_25050	*yqgA*	-3.6	cell wall protein
BSU_30650	*dps*	-3.6	DNA-protecting protein, mini-ferritin
BSU_10200	*yhfE*	-3.6	putative aminopeptidase
BSU_33430	*cysI*	-3.6	assimilatory sulfite reductase (hemoprotein β-subunit)
BSU_14820	*ylaL*	-3.6	conserved hypothetical protein
BSU_39170	*yxzG*	-3.6	putative nucleic acid binding protein
BSU_34330	*epsE*	-3.6	bifunctional flagellar clutch and glycosyltransferase acting during biofilm formation
BSU_33900	*eno*	-3.6	enolase
BSU_26760	*yrdC*	-3.5	putative hydrolase
BSU_17240	*ymzB*	-3.5	conserved protein of unknown function involved in ethanol resistance
BSU_17140	*pksF*	-3.5	bacillaene-related polyketide synthesis
BSU_06450	*purC*	-3.5	phosphoribosylaminoimidazole succinocarboxamide synthetase
BSU_39870	*yxbD*	-3.5	putative N-acetyltransferase
BSU_39160	*yxiI*	-3.5	conserved protein of unknown function
BSU_40590	*yybM*	-3.5	putative transporter permease subunit
BSU_03060	*lctP*	-3.5	L-lactate permease
BSU_21620	*yokE*	-3.4	conserved protein of unknown function, phage SPβ
BSU_17120	*pksE*	-3.4	enzyme involved in bacillaene synthesis
BSU_38080	*ywcI*	-3.4	conserved protein of unknown function
BSU_10170	*fabHB*	-3.4	β-ketoacyl-acyl carrier protein synthase III 2
BSU_23460	*spoIIAB*	-3.4	anti-sigma factor (antagonist of sigma(F)) and serine kinase

BSU_06680	*gatA*	-3.4	glutamyl-tRNA(Gln) amidotransferase (subunit A)
BSU_33320	*fhuD*	-3.4	ferrichrome ABC transporter (ferrichrome-binding lipoprotein)
BSU_29490	*tpx*	-3.4	thiol peroxidase (lipid hydroperoxide reductase)
BSU_39930	*yxaM*	-3.4	putative efflux transporter
BSU_34320	*epsF*	-3.4	putative glycosyltransferase involved in matrix formation (chain-length determination)
BSU_26820	*yrpD*	-3.4	putative lipoprotein
BSU_33040	*fumC*	-3.4	fumarate hydratase
BSU_38090	*vpr*	-3.4	extracellular serine protease
BSU_39150	*yxiJ*	-3.4	conserved protein of unknown function
BSU_30190	*bioI*	-3.4	cytochrome P450 for pimelic acid formation for biotin biosynthesis
BSU_34280	*epsJ*	-3.3	putative glycosyl transferase involved in biofilm matrix formation
BSU_11990	*yjdB*	-3.3	putative exported protein, phage island
BSU_17220	*pksR*	-3.3	trans AT polyketide synthase involved in nonribosomal synthesis of bacillaene
BSU_18340	*ppsA*	-3.3	non-ribosomal plipastatin synthetase A involved in synthesis of plipastatin
BSU_21560	*yokK*	-3.3	conserved protein of unknown function, phage SPβ
BSU_03320	*nasB*	-3.3	assimilatory nitrate reductase (electron transfer subunit NasB)
BSU_08440	*sxzY*	-3.3	xenosiderophore schizokinen (dihydroxamate) transporter binding lipoprotein
BSU_17340	*hfq*	-3.3	Hfq RNA chaperone
BSU_35320	*fliT*	-3.3	flagellar assembly protein FliT involved in control of flagella expression
BSU_22370	*aspB*	-3.3	anabolic oxaloacetate / glutamate aminotransferase
BSU_34240	*epsM*	-3.3	putative O-acetyltransferase involved in biofilm matrix formation
BSU_10030	*hinT*	-3.3	promiscuous Hit-family phosphohydrolase, adenosine phosphoramidase
BSU_39820	*htpG*	-3.3	class III heat-shock protein (ATP-dependent molecular chaperone HSP90)
BSU_14890	*ctaC*	-3.3	cytochrome caa3 oxidase (subunit II)
BSU_37350	*sboA*	-3.3	subtilosin A
BSU_21570	*yokJ*	-3.3	conserved protein of unknown function, phage SPβ
BSU_30200	*bioB*	-3.3	biotin synthase
BSU_22880	*rpfA*	-3.3	RNA degradation presenting factor (ribosomal protein S1 homolog)
BSU_33720	*opuBB*	-3.2	choline ABC transporter (permease)
BSU_31450	*kinB*	-3.2	two-component sensor potassium-binding histidine kinase
BSU_34300	*epsH*	-3.2	putative glycosyltransferase involved in biofilm formation
BSU_38750	*cydB*	-3.2	cytochrome bb' ubiquinol oxidase (subunit II)
BSU_39180	*yxiH*	-3.2	conserved protein of unknown function
BSU_09900	*yhaP*	-3.2	Na+-dependent exporter (ABC permease)
BSU_26826	*yrpDX*	-3.2	expressed polypeptide of unknown function
BSU_31460	*kapB*	-3.2	KinB signal transduction and activation of the phosphorelay to sporulation
BSU_34310	*epsG*	-3.2	biofilm extracellular matrix formation chain-length determining factor
BSU_39139	*yxzJ*	-3.2	conserved protein of unknown function
BSU_23850	*zwf*	-3.2	glucose-6-phosphate 1-dehydrogenase (NADP-dependent)
BSU_10770	*wprA*	-3.2	cell wall-associated protease
BSU_39200	*yxzC*	-3.2	putative nucleic acid binding protein
BSU_10240	*yhfI*	-3.2	putative metal-dependent hydrolase
BSU_23620	*yqkF*	-3.2	NADPH-dependent aldo-keto reductase
BSU_22790	*hbs*	-3.2	non-specific DNA-binding protein HBsu
BSU_40980	*yyaB*	-3.1	putative antibiotic immunity protein
BSU_07350	*vdhT*	-3.1	vanillin dehydrogenase
BSU_39190	*yxiG*	-3.1	conserved hypothetical protein
BSU_06440	*purB*	-3.1	adenylosuccinate lyase
BSU_03050	*lctE*	-3.1	L-lactate dehydrogenase
BSU_24340	*accC*	-3.1	acetyl-CoA carboxylase subunit (biotin carboxylase subunit)
BSU_24620	*tasA*	-3.1	major biofilm matrix component
BSU_10210	*yhfF*	-3.1	putative RNA-binding PUA-containing enzyme
BSU_34265	*epsK*	-3.1	putative extracellular matrix component exporter, putative cyclic di-GMP receptor
BSU_10250	*lplJ*	-3.1	lipoate-protein ligase
BSU_16700	*ylxY*	-3.1	putative sugar deacetylase
BSU_06390	*yebD*	-3.1	hypothetical protein
BSU_23450	*sigF*	-3.1	RNA polymerase sporulation-specific sigma factor (sigma-F)
BSU_10760	*yisL*	-3.0	putative integral membrane protein
BSU_10750	*mblK*	-3.0	hydrolase/isomerase involved in remodelling the bacterial envelope
BSU_14450	*ampS*	-3.0	aminopeptidase
BSU_33440	*cysJ*	-3.0	assimilatory sulfite reductase (flavoprotein alpha-subunit)
BSU_11720	*fabI*	-3.0	enoyl-acyl carrier protein reductase
BSU_30360	*yttA*	-3.0	conserved protein of unknown function
BSU_13040	*hmpA*	-3.0	flavohemoglobin
BSU_25880	*yqxJ*	-3.0	hypothetical protein, skin element

BSU_13010	*pgl*	-3.0	6-phosphogluconolactonase

Table S2. Significantly upregulated and downregulated genes in the VBNC cells compared to the kanamycin sensitive cells (p-value ≤ 0.05 and a logFC ≥ 2)

Locus tag	Gene	Fold change	Function
BSU_03200	*putB*	7.5	proline oxidase
BSU_20520	*yoqT*	7.1	conserved hypothetical protein, phage SPβ
BSU_05408	*ydzP*	6.7	hypothetical protein
BSU_18275	*BSU_18275*	6.6	conserved protein of unknown function
BSU_19900	*yotF*	6.5	hypothetical protein, phage SPβ
BSU_03210	*putC*	6.1	1-pyrroline-5-carboxylate dehydrogenase
BSU_18689	*BSU_18689*	5.9	hypothetical protein
BSU_23616	*mciZ*	5.8	cell division inhibitor
BSU_27570	*yrzK*	5.7	conserved protein of unknown function
BSU_ncRNA_1	*aimX*	4.1	small RNA controlling lysogeny of phage SPβ
BSU_05470	*mneP*	3.7	primary Mn(II) efflux pump
BSU_26020	*yqbP*	3.5	putative phage murein-binding protein, skin element
BSU_20510	*yoqU*	3.4	conserved hypothetical protein, phage SPβ
BSU_03220	*putP*	3.3	proline permease
BSU_03230	*putR*	3.2	transcriptional activator of proline degradation operon
BSU_20950	*yopB*	3.0	putative transcriptional regulator, lambda repressor-like, phage SPβ
BSU_26340	*yqaF*	2.9	putative transcriptional regulator, skin element
BSU_04900	*yddA*	2.9	*ICEBs1* mobile element: conserved protein of unknown function
BSU_10230	*yhfH*	2.8	conserved protein of unknown function
BSU_21420	*bhlA*	2.8	holin-like protein, bacteriophage SPβ
BSU_25875	*BSU_25875*	2.8	hypothetical protein
BSU_13050	*ykzH*	2.8	conserved hypothetical protein
BSU_04910	*conB*	2.6	*ICEBs1* mobile element: subunit of the conjugation machinery VirB8
BSU_26360	*yqaD*	2.6	conserved phage protein of unknown function, skin element
BSU_24090	*ptb*	2.6	phosphate BCFA and butyryl coenzyme A transferase
BSU_13740	*queE*	2.6	7-carboxy-7-deazaguanine synthase
BSU_06320	*mneS*	2.6	secondary Mn(II) exporter, promiscuous
BSU_13720	*queC*	2.5	pre-queuosine 0 synthase
BSU_21410	*blyA*	2.5	bacteriophage SPβ N-acetylmuramoyl-L-alanine amidase
BSU_04890	*ydcT*	2.5	*ICEBs1* mobile element: conserved protein of unknown function
BSU_04920	*conC*	2.5	*ICEBs1* mobile element: subunit of the conjugation machinery
BSU_01880	*ybcI*	2.5	conserved hypothetical protein, prophage 1 region
BSU_13730	*queD*	2.5	6-carboxy-5,6,7,8-tetrahydropterin synthase, queuosine biosynthesis
BSU_04880	*ydcS*	2.5	*ICEBs1* mobile element: conserved protein of unknown function
BSU_21230	*yomT*	2.4	hypothetical protein, phage SPβ
BSU_26935	*BSU_26935*	2.4	hypothetical protein
BSU_12560	*xpf*	2.4	phage PBSX, putative RNA polymerase PBSX sigma factor-like
BSU_19990	*yosV*	2.4	conserved hypothetical protein, phage SPβ
BSU_20070	*nrdIB*	2.4	SPβ phage subunit of ribonucleoside diphosphate reductase
BSU_20550	*yoqP*	2.3	conserved hypothetical protein, phage SPβ
BSU_13750	*queF*	2.3	NADPH-dependent 7-cyano-7-deazaguanine reductase
BSU_20230	*yorW*	2.3	conserved hypothetical protein, phage SPβ
BSU_21170	*yomZ*	2.3	conserved protein of unknown function, phage SPβ
BSU_21030	*yonP*	2.3	hypothetical protein, phage SPβ
BSU_04930	*conD*	2.3	*ICEBs1* mobile element: subunit of the conjugation machinery
BSU_21290	*yomN*	2.3	conserved protein of unknown function, phage SPβ
BSU_20380	*yorH*	2.3	conserved hypothetical protein, phage SPβ
BSU_21270	*yomP*	2.2	conserved phage protein of unknown function, phage SPβ
BSU_01830	*ndhF*	2.2	putative NADH dehydrogenase, prophage 1 region
BSU_21140	*yonC*	2.2	conserved protein of unknown function, phage SPβ
BSU_26827	*BSU_26827*	2.2	hypothetical protein
BSU_24080	*bcd*	2.1	branched-chain amino acid dehydrogenase
BSU_21260	*yomQ*	2.1	putative tail phage assembly protein, phage SPβ
BSU_21160	*yonA*	2.1	conserved protein of unknown function, phage SPβ
BSU_04860	*conQ*	2.1	*ICEBs1* mobile element: coupling conjugation protein VirD4
BSU_20240	*yorV*	2.1	putative nucleic acid binding protein, phage SPβ
BSU_21190	*yomX*	2.1	conserved protein of unknown function, phage SPβ
BSU_20480	*yoqX*	2.1	conserved protein of unknown function, SPβ phage

BSU_14390	*fruK*	2.1	fructose-1-phosphate kinase
BSU_24070	*buk*	2.1	branched-chain fatty-acid kinase
BSU_26619	*yrzO*	2.1	conserved protein of unknown function
BSU_04980	*yddI*	2.0	*ICEBs1* mobile element: conserved protein of unknown function
BSU_20280	*yorR*	2.0	putative nucleotide kinase or triphosphate hydrolase, phage SPβ
BSU_04870	*nicK*	2.0	*ICEBs1* mobile element: DNA relaxase
BSU_37360	*sboX*	-6.4	putative bacteriocin-like product
BSU_misc_RNA_75	*bsrF*	-5.7	
BSU_11799	*yjzK*	-4.6	conserved hypothetical protein
BSU_32990	*mrgA*	-4.0	metalloregulation DNA-binding stress protein
BSU_27030	*sacC*	-3.9	levanase
BSU_35950	*rbsC*	-3.9	ribose ABC transporter (permease)
BSU_37180	*fadF*	-3.8	putative iron-sulphur-binding reductase
BSU_32830	*fadA*	-3.8	acetyl-CoA C-acyltransferase
BSU_33410	*yvgO*	-3.8	exported stress induced factor
BSU_04400	*gsiB*	-3.8	general stress protein glucose starvation induced
BSU_27070	*levD*	-3.8	phosphotransferase system (PTS) fructose-specific enzyme IIA component
BSU_35960	*rbsB*	-3.7	ribose ABC transporter (ribose-binding lipoprotein)
BSU_40660	*yybF*	-3.7	putative permease
BSU_32820	*fadE*	-3.7	acyl-CoA dehydrogenase (FAD dependent)
BSU_38560	*licH*	-3.7	6-phospho-beta-glucosidase
BSU_36670	*csbD*	-3.7	stress response protein
BSU_38580	*licC*	-3.6	phosphotransferase system (PTS) lichenan-specific enzyme IIC component
BSU_35940	*rbsA*	-3.6	ribose ABC transporter (ATP-binding protein)
BSU_38570	*licA*	-3.6	phosphotransferase system (PTS) lichenan-specific enzyme IIA component
BSU_34920	*hisG*	-3.6	ATP phosphoribosyltransferase
BSU_08820	*katA*	-3.6	vegetative catalase 1
BSU_34910	*hisD*	-3.5	histidinol dehydrogenase
BSU_27050	*levF*	-3.5	phosphotransferase system (PTS) fructose-specific enzyme IIC component
BSU_35920	*rbsK*	-3.5	ribokinase
BSU_32840	*fadN*	-3.4	bifunctional enoyl-CoA hydratase / 3-hydroxyacyl-CoA dehydrogenase
BSU_27040	*levG*	-3.4	phosphotransferase system (PTS) fructose-specific enzyme IID component
BSU_39810	*csbC*	-3.4	putative metabolite transporter
BSU_34890	*hisH*	-3.4	imidazole glycerol phosphate synthase, glutamine amidotransferase subunit
BSU_35910	*rbsR*	-3.4	ribose operon repressor (LacI family, D-ribose)
BSU_27060	*levE*	-3.3	phosphotransferase system (PTS) fructose-specific enzyme IIB component
BSU_33240	*oxdC*	-3.3	oxalate decarboxylase
BSU_40070	*gntP*	-3.3	gluconate/proton permease
BSU_35970	*ywsB*	-3.3	putative cell wall binding enzyme
BSU_34900	*hisB*	-3.3	imidazoleglycerol-phosphate dehydratase [Mn(II)-dependent]
BSU_13160	*ohrB*	-3.2	organic hydroperoxide resistance reductase B
BSU_33810	*opuCC*	-3.2	glycine betaine/carnitine/choline/choline sulfate ABC transporter (osmoprotectant-binding lipoprotein)
BSU_35930	*rbsD*	-3.2	D-ribose pyranase
BSU_33820	*opuCB*	-3.2	glycine betaine/carnitine/choline/choline sulfate ABC transporter (promiscuous permease)
BSU_04380	*ydaT*	-3.2	conserved protein of unknown function
BSU_02110	*ybyB*	-3.2	conserved protein of unknown function
BSU_40060	*gntK*	-3.2	D-gluconate kinase
BSU_08570	*yfhK*	-3.2	putative exported protein
BSU_34930	*hisZ*	-3.1	histidyl-tRNA synthetase-like subunit of ATP phophoribosyltransferase
BSU_34870	*hisF*	-3.1	imidazole glycerol phosphate synthase subunit
BSU_38590	*licB*	-3.1	phosphotransferase system (PTS) lichenan-specific enzyme IIB component
BSU_39840	*yxbG*	-3.1	putative oxidoreductase
BSU_33830	*opuCA*	-3.1	glycine betaine/carnitine/choline/choline sulfate ABC transporter (ATP-binding protein)
BSU_37170	*acdA*	-3.1	acyl-CoA dehydrogenase
BSU_30260	*msmR*	-3.1	transcriptional regulator (LacI family)
BSU_33800	*opuCD*	-3.1	glycine betaine/carnitine/choline/choline sulfate ABC transporter (promiscuous permease)
BSU_03320	*nasB*	-3.1	assimilatory nitrate reductase (electron transfer subunit NasB)
BSU_04730	*sigB*	-3.0	RNA polymerase sigma-37 factor (sigma(B))
BSU_04740	*rsbX*	-3.0	serine phosphatase
BSU_07550	*yflT*	-3.0	heat stress induced protein
BSU_38810	*msmX*	-3.0	multiple sugar (maltodextrins) transporter ATP-binding protein
BSU_34860	*hisIE*	-3.0	bifunctional phosphoribosyl-AMP cyclohydrolase, phosphoribosyl-ATP pyrophosphohydrolase

BSU_39050	*katE*	-3.0	catalase 2
BSU_33239	*yvrJ*	-3.0	factor involved in oxalate decarboxylase expression
BSU_28780	*araD*	-3.0	L-ribulose-5-phosphate 4-epimerase
BSU_27786	*BSU_27786*	-3.0	hypothetical protein
BSU_04370	*ydaS*	-2.9	conserved protein of unknown function
BSU_07920	*chaA*	-2.9	H+/Ca2+ antiporter
BSU_04360	*mntH*	-2.9	proton-coupled manganese transporter
BSU_34880	*hisA*	-2.9	phosphoribosylformimino-5-aminoimidazole carboxamide ribotide isomerase
BSU_04340	*poxB*	-2.9	acetyl-phosphate generating pyruvate oxidase
BSU_27035	*BSU_27035*	-2.9	hypothetical protein
BSU_40100	*ahpF*	-2.8	alkyl hydroperoxide reductase (large subunit)
BSU_24770	*mgsR*	-2.8	transcriptional regulator of stress
BSU_18520	*yoxB*	-2.8	conserved protein of unknown function
BSU_33230	*sigO*	-2.8	alternative sigma factor
BSU_40000	*yxnA*	-2.8	putative oxidoreductase
BSU_30280	*msmF*	-2.8	carbohydrate ABC transporter (permease)
BSU_28770	*araL*	-2.8	glycolytic and pentose phosphate intermediates phosphatase
BSU_07750	*yflA*	-2.8	putative aminoacid transporter
BSU_09530	*akrN*	-2.8	aldo/keto reductase specific for NADPH, protects against methylglyoxal
BSU_13850	*pfeT*	-2.7	Fe(II) efflux transporter possibly involved in copper(II) uptake
BSU_03780	*phrC*	-2.7	secreted regulator of the activity of phosphatase RapC and competence and sporulation stimulating factor (CSF)
BSU_38180	*ywzA*	-2.7	conserved protein of unknown function
BSU_39270	*bglP*	-2.7	phosphotransferase system (PTS) beta-glucoside-specific enzyme IIBCA component
BSU_33222	*rsoA*	-2.7	regulator of sigma-O
BSU_28520	*etfA*	-2.7	electron transfer flavoprotein (alpha subunit)
BSU_04039	*yczO*	-2.7	conserved protein of unknown function
BSU_29780	*ytxG*	-2.7	general stress protein
BSU_30270	*msmE*	-2.7	multiple sugar-binding lipoprotein
BSU_38240	*vbfA*	-2.7	acetate Na+-dependent symporter subunit involved in volatile signal for biofilm formation
BSU_28760	*egsA*	-2.6	sn-glycerol-1-phosphate dehydrogenase [NAD+] (catabolic)
BSU_28810	*abnA*	-2.6	arabinan-endo-1,5-alpha-L-arabinase
BSU_12170	*yjgD*	-2.6	conserved hypothetical protein
BSU_09870	*khtS*	-2.5	K+/H+ antiporter for K+ efflux
BSU_29770	*ytxH*	-2.5	conserved protein of unknown function
BSU_28750	*araN*	-2.5	sugar-binding lipoprotein
BSU_07800	*treP*	-2.5	phosphotransferase system (PTS) trehalose-specific enzyme IIBC component
BSU_12160	*yjgC*	-2.5	putative molybdoenzyme, putative formate dehydrogenase
BSU_07900	*rbn*	-2.5	putative ribonuclease BN
BSU_02840	*ycdG*	-2.5	putative oligo-carbohydrate hydrolase
BSU_02830	*ycdF*	-2.5	putative oxidoreductase
BSU_09690	*nhaX*	-2.5	stress response protein, UspA family
BSU_39260	*bglH*	-2.5	aryl-phospho-beta-d-glucosidase
BSU_30290	*msmG*	-2.5	maltose and multiple sugars ABC transporter (permease)
BSU_40090	*ahpC*	-2.4	alkyl hydroperoxide reductase (small subunit)
BSU_33940	*gapA*	-2.4	glyceraldehyde-3-phosphate dehydrogenase (NAD-dependent, glycolytic)
BSU_38430	*gspA*	-2.4	putative glycosyl transferase (general stress protein)
BSU_29760	*brxJ*	-2.4	bacilliredoxin involved in debacillithiolation
BSU_40050	*gntR*	-2.4	transcriptional regulator (GntR-gluconate)
BSU_18380	*iseA*	-2.4	inhibitor of DL-endopeptidases involved in cell-separation
BSU_39400	*pdp*	-2.4	pyrimidine-nucleoside phosphorylase
BSU_40080	*gntZ*	-2.4	6-phosphogluconate dehydrogenase (NAD+-dependent)
BSU_10270	*lcfB*	-2.4	long-chain fatty-acid-CoA ligase (degradative)
BSU_14060	*fadH*	-2.4	putative 2,4-dienoyl-CoA reductase
BSU_09860	*khtT*	-2.4	K+/H+ antiporter for K+ efflux
BSU_14590	*pdhB*	-2.4	pyruvate dehydrogenase (E1 beta subunit)
BSU_07930	*yfkD*	-2.4	conserved hypothetical protein
BSU_33900	*eno*	-2.4	enolase
BSU_09850	*khtU*	-2.4	proton/potassium antiporter, methylglyoxal resistance
BSU_28740	*araP*	-2.4	arabinose/arabinan permease
BSU_28790	*araB*	-2.4	L-ribulokinase
BSU_03990	*mtlD*	-2.4	mannitol-1-phosphate 5-dehydrogenase
BSU_33910	*pgm*	-2.3	phosphoglycerate mutase
BSU_33420	*nhaK*	-2.3	Na+/H+ antiporter
BSU_07880	*yfkJ*	-2.3	protein-tyrosine-phosphatase

BSU_08200	*malP*	-2.3	phosphotransferase system (PTS) maltose-specific enzyme IICB component
BSU_28540	*fadB*	-2.3	enoyl-CoA hydratase
BSU_30300	*melA*	-2.3	alpha-D-galactoside galactohydrolase
BSU_15100	*ylbP*	-2.3	putative acetyltransferase
BSU_28720	*abfA*	-2.3	alpha-L-arabinofuranosidase
BSU_08100	*acoR*	-2.3	transcriptional regulator (AcoR-acetoin)
BSU_35080	*pchR*	-2.3	transcriptional regulator for pulcherriminic acid synthesis
BSU_04190	*ydaD*	-2.3	putative dehydrogenase
BSU_03300	*nasD*	-2.3	assimilatory nitrite reductase subunit
BSU_39250	*yxiE*	-2.3	phosphate starvation protein (universal stress protein A family)
BSU_07810	*treA*	-2.3	trehalose-6-phosphate hydrolase
BSU_14580	*pdhA*	-2.2	pyruvate dehydrogenase (E1 alpha subunit)
BSU_28730	*araQ*	-2.2	arabinose/arabinan permease
BSU_07850	*sufLB*	-2.2	general stress protein 18, deglycase
BSU_40110	*bglA*	-2.2	aryl-6-phospho-beta-glucosidase
BSU_03981	*mtlA*	-2.2	phosphotransferase system (PTS) mannitol-specific enzyme IICB component
BSU_28550	*fadR*	-2.2	transcriptional regulator of fatty acids degradation
BSU_39040	*yxiS*	-2.2	conserved protein of unknown function
BSU_18510	*yoxC*	-2.2	transition-dependent and sulfur-related metabolism protein
BSU_28530	*etfB*	-2.2	electron transfer flavoprotein (beta subunit)
BSU_09880	*yhaR*	-2.2	putative dehydratase / isomerase
BSU_33920	*tpiA*	-2.2	triose phosphate isomerase
BSU_04720	*rsbW*	-2.2	switch protein/serine kinase and anti-sigma factor
BSU_39720	*iolE*	-2.2	scyllo-inosose dehydratase
BSU_10430	*yhxD*	-2.2	putative oxidoreductase
BSU_39690	*iolH*	-2.2	putative sugar-phosphate epimerase/isomerase
BSU_31340	*yugM*	-2.2	putative transporter
BSU_07890	*yfkI*	-2.2	conserved hypothetical protein
BSU_35090	*pchE*	-2.2	pulcherriminic acid efflux transporter
BSU_28710	*cstA*	-2.2	carbon starvation-induced membrane protein
BSU_34190	*lutP*	-2.2	L-lactate permease
BSU_38630	*katX*	-2.2	major catalase in spores
BSU_misc_RNA_82	*surF*	-2.1	
BSU_27770	*yrbE*	-2.1	putative inositol-related oxidoreductase
BSU_33930	*pgk*	-2.1	phosphoglycerate kinase
BSU_07050	*rhgW*	-2.1	rhamnogalacturonan endolyase
BSU_03385	*BSU_03385*	-2.1	hypothetical protein
BSU_09140	*yhcM*	-2.1	expressed protein of unknown function
BSU_06560	*yerA*	-2.1	putative adenine deaminase YerA
BSU_06590	*yerD*	-2.1	putative osmotic shock glutamate synthase subunit
BSU_12900	*htrA*	-2.1	membrane bound serine protease Do, quality control protease and chaperone
BSU_40450	*yycD*	-2.1	conserved hypothetical protein
BSU_34150	*ganP*	-2.1	galacto-oligosaccharides (galactan) oligomer permease
BSU_39410	*nupC*	-2.1	pyrimidine-nucleoside Na+(H+) cotransporter
BSU_38190	*galT*	-2.1	galactose-1-phosphate uridyltransferase
BSU_34120	*ganB*	-2.1	endo-beta-1,4-galactanase
BSU_30650	*dps*	-2.1	DNA-protecting protein, mini-ferritin
BSU_03060	*lctP*	-2.1	L-lactate permease
BSU_33400	*pgoN*	-2.1	promiscuous glyoxal/methylglyoxal reductase
BSU_34140	*ganQ*	-2.1	galacto-oligosaccharides (galactan) oligomer permease
BSU_03310	*nasC*	-2.1	assimilatory nitrate reductase (catalytic subunit)
BSU_08110	*sspH*	-2.1	small acid-soluble spore protein
BSU_33950	*cggR*	-2.0	transcriptional regulator of gapA
BSU_38610	*yxzF*	-2.0	conserved protein of unknown function
BSU_39710	*iolF*	-2.0	inositol transport protein
BSU_34160	*ganS*	-2.0	polygalactose / cyclodextrin-binding lipoprotein
BSU_24760	*rsbRD*	-2.0	component of the anxiosome (stressosome)
BSU_21940	*degR*	-2.0	activator of degradative enzymes (aprE, nprE, sacB) production or activity
BSU_11500	*spxA*	-2.0	redox-sensitive regulator
BSU_27760	*csbX*	-2.0	putative catecholate siderophose exporter
BSU_34130	*ganA*	-2.0	short chain beta-1,4-galacto-oligosaccharides beta-galactosidase (beta-galacto-pyranoside hydrolase)
BSU_07070	*yesY*	-2.0	rhamnogalacturonan acetylesterase
